# Sex separation induces differences in the olfactory sensory receptor repertoires of male and female mice

**DOI:** 10.1038/s41467-018-07120-1

**Published:** 2018-12-04

**Authors:** Carl van der Linden, Susanne Jakob, Pooja Gupta, Catherine Dulac, Stephen W. Santoro

**Affiliations:** 1 Neuroscience Program, Department of Zoology & Physiology, University of Wyoming, 1000 E. University Avenue, Laramie, WY 82071 USA; 2 Howard Hughes Medical Institute, Department of Molecular and Cellular Biology, Center for Brain Science, Harvard University, 16 Divinity Avenue, Cambridge, MA 02138 USA; 3 Present Address: Department of Stem Cell and Regenerative Biology, Harvard University, 7 Divinity Avenue, Cambridge, MA 02138 USA

## Abstract

Within the mammalian olfactory sensory epithelium, experience-dependent changes in the rate of neuronal turnover can alter the relative abundance of neurons expressing specific chemoreceptors. Here we investigate how the mouse olfactory sensory receptor repertoire changes as a function of exposure to odors emitted from members of the opposite sex, which are highly complex and sexually dimorphic. Upon housing mice either sex-separated or sex-combined until six months of age, we find that sex-separated mice exhibit significantly more numerous differentially expressed genes within their olfactory epithelia. A subset of these chemoreceptors exhibit altered expression frequencies following both sex-separation and olfactory deprivation. We show that several of these receptors detect either male- or female-specific odors. We conclude that the distinct odor experiences of sex-separated male and female mice induce sex-specific differences in the abundance of neurons that detect sexually dimorphic odors.

## Abstract (web summary)

Olfactory experience can alter the relative abundance of neurons expressing specific chemoreceptors. Here, the authors demonstrate that the distinct odor experiences of sex-separated male and female mice induce sex-specific differences in the abundance of neurons that detect sexually dimorphic odors.

## Introduction

Sensory activity plays pivotal roles in shaping the development of the nervous system, as revealed in early studies of monocular light deprivation, which was found to severely disrupt the formation of ocular dominance columns in the visual system^1^. More recently, novel insights into molecular mechanisms underlying activity-dependent neuronal plasticity have led to a better understanding of these phenomena and their involvement in neurodevelopmental disorders^2^. In the olfactory system, activity plays important roles in both the formation and the refinement of precise connections between olfactory sensory neurons (OSNs) located in the main olfactory epithelium (MOE) and projection neurons within the olfactory bulb (OB)^3–8^. Moreover, olfactory experience modulates the abundance of specific OSN subtypes, as defined by the single olfactory receptor (OR) gene that each OSN expresses^9–14^. These changes appear to occur through modulation of the lifespan of distinct OSN subtypes. Unlike most neurons in the mammalian nervous system, OSNs are continually born and replaced throughout life^15^. Changes in the abundance of specific OSN subtypes occur in part through a use-it-or-lose-it-type mechanism, in which active OSNs are retained and silent OSNs are eliminated from the population^10–13^. Additionally, some changes in OSN subtype abundance appear to be mediated by a use-it-and-lose-it-type mechanism, in which odor stimulation, perhaps exceeding a threshold level, selectively reduces the abundance of specific OSN subtypes. Evidence for the latter mechanism comes from findings that the abundance of some OSN subtypes is selectively increased following olfactory deprivation^10,11,13^ or reduced following stimulation by specific odors^9,14^. Moreover, the relative expression levels of OR subtypes change with age^11,16–19^, consistent with the idea that the OSN population is plastic. Vomeronasal sensory neurons (VSNs), like OSNs, also undergo turnover throughout life^20^, suggesting that the abundance of VSN subtypes may have a similar capacity for experience-dependent modulation. Interestingly, studies have revealed that the prolonged exposure of male mice to a specific ligand found in female urine drastically reduces the physiological responsiveness of VSNs to the ligand^21^, but whether this phenomenon involves changes in the abundance of VSNs or simply their silencing is unknown. The precise physiological role of activity-dependent changes to the representation of OSN subtypes remains to be determined but has been hypothesized to play a role in adapting an individual’s olfactory system to the detection and/or discrimination of salient odors, which may vary from one olfactory environment to another^11^.

Studies to date indicate that changes in the relative numbers of OSN subtypes within the MOE require neuronal turnover and thus occur on a timescale of weeks to months^11,14^. Notably, these changes appear to be distinct from a phenomenon that occurs on a timescale of hours that entails changes in the number of OR transcripts expressed within individual OSNs but not in the abundance of specific OSN subtypes^22^. Likewise, these changes appear distinct from a phenomenon involving the rapid and temporary loss of responsiveness of a subset of female VSNs to specific male pheromones as a result of progesterone signaling during diestrus^23^.

Here we have sought to investigate olfactory system plasticity as a function of mouse exposure to, or isolation from, odors from the opposite sex for a prolonged time period. Mouse odors are complex mixtures of volatile and non-volatile chemicals derived from skin secretions, urine, tears, saliva, and feces, which are known to differ substantially in their chemical composition between males and females^24–32^. Indeed odors emitted by males and females have been shown to activate distinct subsets of OSNs and VSNs^21,24,26,27,32–39^. Sex-specific odors facilitate behaviors that are critical for species survival, such as mating and aggression, and are thus highly behaviorally salient^40,41^. Here we have housed male and female mice either separated from members of the opposite sex (sex-separated) or combined with members of the opposite sex (sex-combined) from weaning until 6 months of age. Because male and female mice emit distinct odor profiles, we predicted that sex-separated males and females (SM and SF, respectively) would have distinct olfactory experiences and would therefore display differences in their profiles of OSN subtypes and overall olfactory gene expression. By contrast, sex-combined male and female (CM and CF, respectively) mice are likely to have highly similar olfactory experiences and should thus display fewer differences in their profiles of OSN subtypes and gene expression. To test these predictions, we analyzed the MOE, VNO, and OB tissues from sex-separated and sex-combined mice via RNA-seq and histology.

Here we report that the expression frequencies of specific chemoreceptors, as well as overall gene expression, are significantly more divergent in the MOE and VNO organs of SM and SF compared to their sex-combined counterparts. Moreover, we show that OSNs that express affected chemoreceptors are activated by exposure to either male or female mice and are altered in abundance by olfactory sensory deprivation. Our findings suggest that differences in the odor environments of SM and SF mice induce sex-specific differences in the olfactory neuron population, likely as a result of altered rates of neuron turnover. Results from this study may contribute to an enhanced understanding of sex-specific differences in olfactory function^41^.

## Results

### Sex separation alters gene expression sex-specifically

**Fig. 1:**
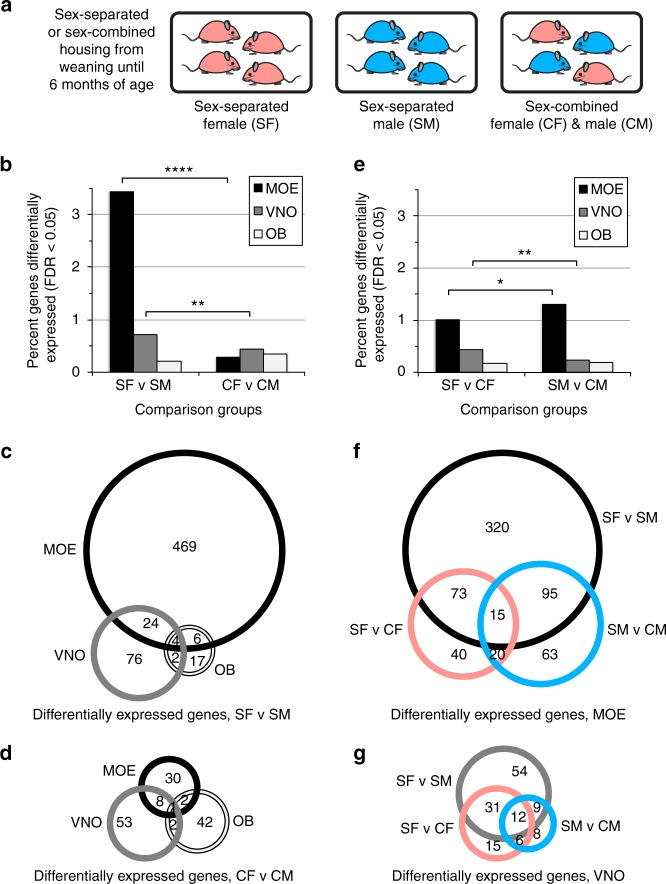
Sex separation induces gene expression differences between male and female mice in MOE and VNO tissues. **a** Experimental set-up. From weaning (P21) until 6 months of age, mice experienced either a sex-separated environment, in which mice were housed either 4 females/cage (left) or 4 males/cage (middle), or a sex-combined environment (right), in which mice were housed 2 females+2 males/cage. **b** Percentage of expressed genes that were identified via RNA-seq as significantly differentially expressed (FDR < 0.05) within MOE, VNO, and OB tissues between male and female sex-separated (SF versus SM) and sex-combined (CF versus CM) mice. **c**, **d** Venn diagram of expressed genes identified via RNA-seq as significantly differentially expressed (FDR < 0.05) within MOE, VNO, and OB tissues between male and female sex-separated (SF versus SM) (**c**) and sex-combined (CF versus CM) (**d**) mice. **e** Percentage of expressed genes that were identified via RNA-seq as significantly differentially expressed (FDR < 0.05) within MOE, VNO, and OB tissues between sex-separated and sex-combined female (SF versus CF) and male (SM versus CM) mice. **f**, **g** Venn diagram of expressed genes identified via RNA-seq as significantly differentially expressed (FDR < 0.05) within MOE, VNO, and OB tissues between sex-separated and sex-combined female (SF versus CF) (**f**) and male (SM versus CM) (**g**) mice. SF sex-separated females, SM sex-separated males, CF sex-combined females, SM sex-combined males. *p < 0.05, **p < 0.01, ****p <0.0001 (Chi-squared test)

We sought to test whether olfactory tissues of male and female mice housed separately from members of the opposite sex (sex-separated) for an extended period of time would display greater differences in the expression of chemoreceptors and other genes compared to males and females housed together (sex-combined). By housing four females, four males, or two females and two males per cage from weaning (postnatal day 21 (PD 21)) until 6 months of age, we generated SF, SM, or CF and CM, respectively (Fig. 1a; Supplementary Fig. 1; Methods). Based on previous studies^11^, we predicted that the >5-month period of sex separation would be of sufficient length for experience-dependent changes in OSN subtype abundance to arise. Upon weaning, SF and SM cages were transferred to rooms containing only mice of the same sex to further minimize exposure to opposite-sex odors. Pups born in the sex-combined cages were euthanized within 24 h of birth in order to minimize exposure to pup-specific odors. We observed no signs of aggression in any of the mouse groups, consistent with previous findings that multiple male mice of the C57Bl/6 strain can cohabitate indefinitely into adulthood without fighting if consistently kept together from the time of weaning^42^. At 6 months of age, MOE, VNO, and OB tissues were removed from all mice.

Using RNA-seq analysis of total RNA, we profiled gene expression in the MOE, VNO, and OB tissues from all the four experimental groups (Supplementary Data 1-3)^43^. For each tissue, we first sought to compare the number of genes differentially expressed (false discovery rate (FDR) < 0.05) between males and females under sex-separated versus sex-combined conditions. In the MOE, a remarkable 3.4% of all genes were differentially expressed between SF and SM mice, 12-fold higher than the 0.28% of genes differentially expressed between CF and CM mice (504 versus 42 genes; *p* < 0.0001; N-1 Chi-squared test) (Fig. 1b). In the VNO, a relatively modest but still significant 1.7-fold larger percentage of genes were differentially expressed between SF and SM mice than between CF and CM mice (0.72% versus 0.44% of expressed genes; 106 versus 64 genes; *p* = 0.0016; N-1 Chi-squared test). In the OB, interestingly, we actually observed a 1.6-fold *smaller* number of genes that were differentially expressed between SF and SM mice than between CF and CM mice (0.21% versus 0.35% of expressed genes; 29 versus 47 genes; *p* = 0.03; N-1 Chi-squared test). Comparing the number of genes differentially expressed between sex-separated and sex-combined males and females [(SF versus SM)–(CF versus CM)] in each tissue, we found that the MOE exhibited a significantly larger divergence in the number of genes altered in their expression under the two conditions compared to the VNO (462 more genes versus 42 more; *p* < 0.0001; N-1 Chi-squared test) or the OB (462 more genes versus 18 fewer; *p* < 0.0001; N-1 Chi-squared test). These data indicate that sex separation induces numerous gene expression differences between males and females in the MOE and VNO and that the MOE displays an especially high level of plasticity based on environmental conditions.

For each experimental condition, we next looked for common genes among those differentially expressed between male and female mice in the MOE, VNO, and OB. In the SF versus SM comparison, the MOE and VNO had 28 differentially expressed genes in common, the MOE and OB had 10, the VNO and OB had 6, and all three tissues had 4 (Fig. 1c; Supplementary Data 4). In the CF versus CM comparison, the MOE and VNO shared 9 common differentially expressed genes, the MOE and OB shared 3, the VNO and OB shared 3, and all three tissues shared 1 (Fig. 1d; Supplementary Data 5). These results indicate that, while sex separation may affect the expression of some common genes in the MOE and VNO, the identity of genes differentially expressed between males and females is largely specific to tissue and condition. Notably, three of the four genes that displayed expression differences between males and females in all three tissues (the X-linked *Kdm6a* and the Y-linked *Kdm5d* and *Ddx3y* genes) have been reported to exhibit sex-specific expression in multiple tissues^44^. The fourth gene (*Gm14743*) encodes an odorant-binding protein shown to be differentially expressed in the MOE between male and female mice^45^.

Gene expression differences observed in the MOE and VNO between SF and SM mice could arise from changes predominantly within one sex or via expression changes in both sexes. To investigate these alternative scenarios, we compared the number of significant gene expression differences between sex-separated and sex-combined females (SF versus CF) and between sex-separated and sex-combined males (SM versus CM). In the MOE, the SF versus CF and SM versus CM comparisons revealed 148 and 193 differentially expressed genes, respectively (1.0% and 1.3% of expressed genes; Fig. 1e; *p* = 0.0127; N-1 Chi-squared test). In the VNO, the SF versus CF and SM versus CM comparisons revealed 64 and 35 differentially expressed genes (0.46% and 0.25%; *p* = 0.0025; N-1 Chi-squared test), respectively. In both the MOE and VNO, the identities of differentially expressed genes in the SF versus CF and SM versus CM comparisons exhibited substantial overlap (Fig. 1f, g; Supplementary Data 6, 7). Taken together, these results indicate that both sexes undergo changes in gene expression due to sex-separation, with males exhibiting a greater number of changes in the MOE and females exhibiting a greater number of changes in the VNO.

### Sex separation alters OR expression in the MOE

**Fig. 2:**
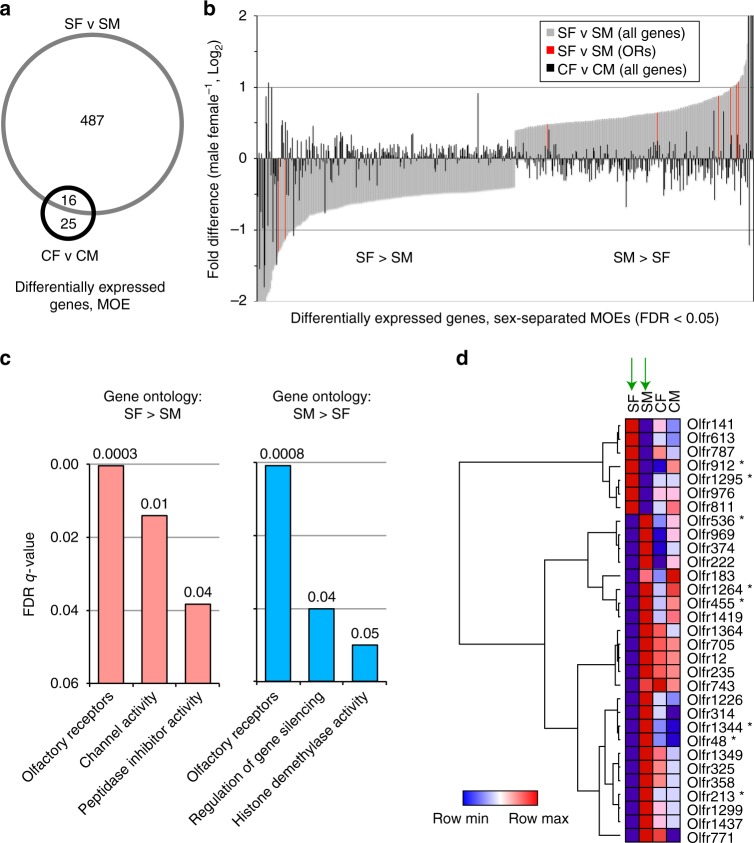
Sex separation induces sex-specific differences in the expression of genes, including ORs, in the MOE. **a** Venn diagram of expressed genes identified via RNA-seq as significantly differentially expressed (FDR < 0.05) within the MOE between male and female sex-separated (SF versus SM) and sex combined (CF versus CM) mice. **b** Comparison of the differential expression of individual genes within the MOE between SF and SM mice (gray) or between SF and CM mice (black). Shown are genes identified via RNA-seq as significantly differentially expressed (FDR < 0.05) in the MOE between SF and SM mice. Genes encoding odorant receptors (ORs) are highlighted in red. **c** Enriched gene ontology (GO) categories among genes identified by RNA-seq as significantly differentially expressed (FDR < 0.05) in the MOE between SF and SM mice. Shown are analyses of female-biased (SF > SM; left) and male-biased (SM > SF; right) genes. **d** Hierarchical clustering of OR-encoding genes identified via RNA-seq as significantly differentially expressed between SF and SM mice (green arrows). ORs labeled asterisk, FDR < 0.05; other ORs shown, unadjusted *p* < 0.01 (Cufflinks output). SF sex-separated females, SM sex-separated males, CF sex-combined females, SM sex-combined males

Considering their striking abundance, we next sought to characterize genes found to exhibit differential expression in the MOE between SF and SM mice. Remarkably, 97% (487 of 503) of these genes were differentially expressed only under sex-separated conditions (i.e., not between CF and CM mice) (Fig. 2a, b; Supplementary Data 8). Gene ontology (GO) analyses of genes significantly differentially expressed (FDR < 0.05) in the MOE between SF and SM mice revealed ORs as the category most significantly enriched among both male-biased and female-biased genes (Fig. 2c). Similar to overall gene expression, the number of ORs differentially expressed was four-fold greater between SF and SM mice than between CF and CM mice (31 versus 8 ORs; 4.4% versus 1.1% of ORs meeting the minimum expression criteria; *p* = 0.0001; N-1 Chi-squared test; Supplementary Fig. 2a). Moreover, sex-separation-induced changes in OR expression appear to have arisen via changes within both sexes, with a slightly larger contribution from changes in males (Supplementary Fig. 2a–d). Notably, the identities of ORs differentially expressed between SF and SM mice were completely distinct from those differentially expressed between CF and CM mice (Fig. 2d; Supplementary Fig. 2e), underscoring that the environment is a critical determinant of sex-biased OR expression.

### OR expression differences reflect changes in OSN abundance

**Fig. 3:**
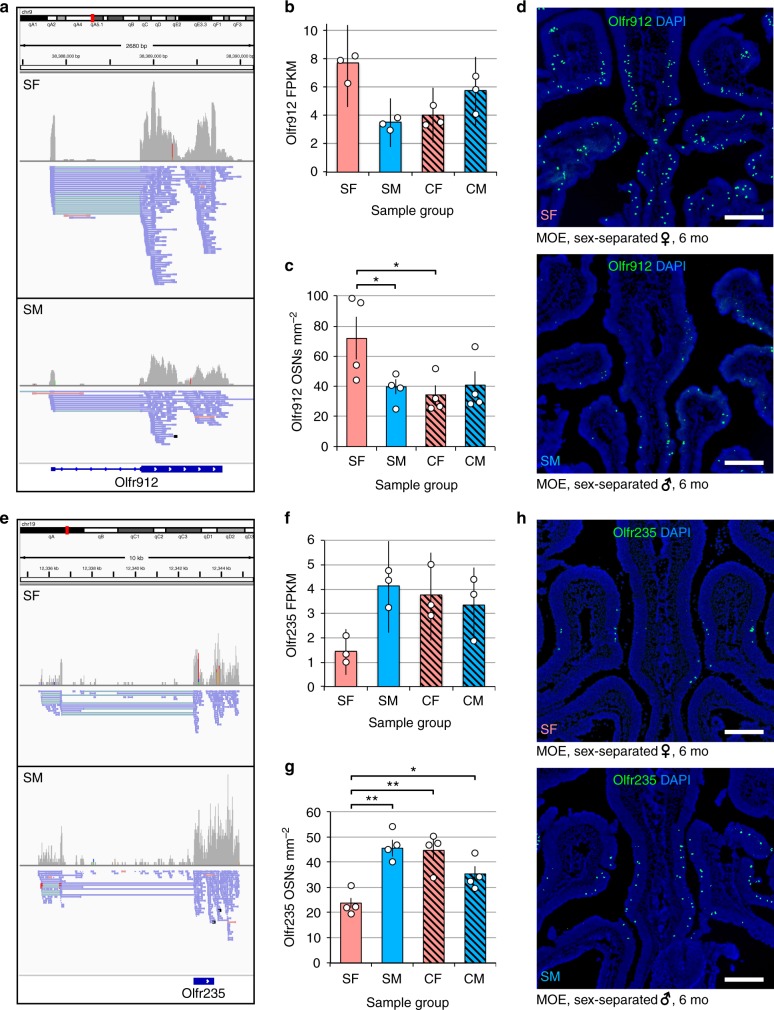
Differences in OR expression between SF and SM mice reflect differences in the abundance of corresponding OSN subtypes, as revealed for two representative ORs. **a**, **e** RNA-seq read alignments to *Olfr912* (**a**) and *Olfr235* (**e**) for SF and SM samples. For simplicity, alignments from the three biological replicates in each experimental group were combined (SF, top; SM; bottom). Strand orientation is indicated by color: − strand, pink; + strand, blue. **b**, **f** Expression levels determined by RNA-seq FPKM values for *Olfr912* (**b**) and *Olfr235* (**f**) in the MOEs of the experimental mouse groups shown. Error bars: 95% c.i. **c**, **g** Quantification, using two-color RNA-FISH, of the frequency of *Olfr912*-expressing (**c**) and *Olfr235*-expressing (**g**) OSNs relative to the area of all mature OSNs (based on *Omp* expression) for each experimental group. Error bars: s.e.m. *p < 0.05, **p < 0.01 (two-tailed *t* test); *n* = 4 mice (5 sections/mouse; average of 41 *Olfr912*-expressing and 32 *Olfr235*-expressing OSNs/section). Dots represent average values for individual mice. **d**, **h** Representative images of *Olfr912* (**d**) and *Olfr235* (**h**) expression in the MOEs of the experimental mouse groups shown. SF sex-separated females, SM sex-separated males, CF sex-combined females, SM sex-combined males. Scale bars: 400 µm

Observed OR expression differences may reflect differences either in the number of OSNs expressing a given OR gene^11,14,16,46^ or in the number of OR transcripts within individual OSNs^22,46,47^. To test these alternative scenarios, we chose 8 representative ORs found to be significantly differentially expressed by RNA-seq in SF and SM mice and quantified their expression frequencies within coronal sections of MOE from SF, SM, CF, and CM mice by fluorescent in situ hybridization (FISH). Four of the 8 ORs analyzed (*Olfr912*, *Olfr235*, *Olfr1419*, and *Olfr1437*) were found to exhibit differences in expression frequencies that closely paralleled differences in OR mRNA transcript levels determined by RNA-seq (Fig. 3; Supplementary Figs. 2f, 3). By contrast, the remaining four ORs (*Olfr141*, *Olfr1295*, *Olfr976*, and *Olfr48*) did not show statistically significant differences in expression frequencies (Supplementary Fig. 2f). The latter result suggests that some of the OR expression differences observed by RNA-seq may not reflect differences in the numbers of OSNs expressing affected ORs. However, an additional or alternative potential explanation is that some of the FISH probes used likely detect more than one type of OR transcript (Supplementary Table 1), in which case differences in the expression frequency of the target OR could be masked. Nonetheless, these results indicate that at least approximately half of the OR expression differences between SF and SM mice observed by RNA-seq reflect differences in the numbers of OSN subtypes in which the affected ORs are expressed.

### Differentially expressed ORs detect sexually dimorphic odors

**Fig. 4:**
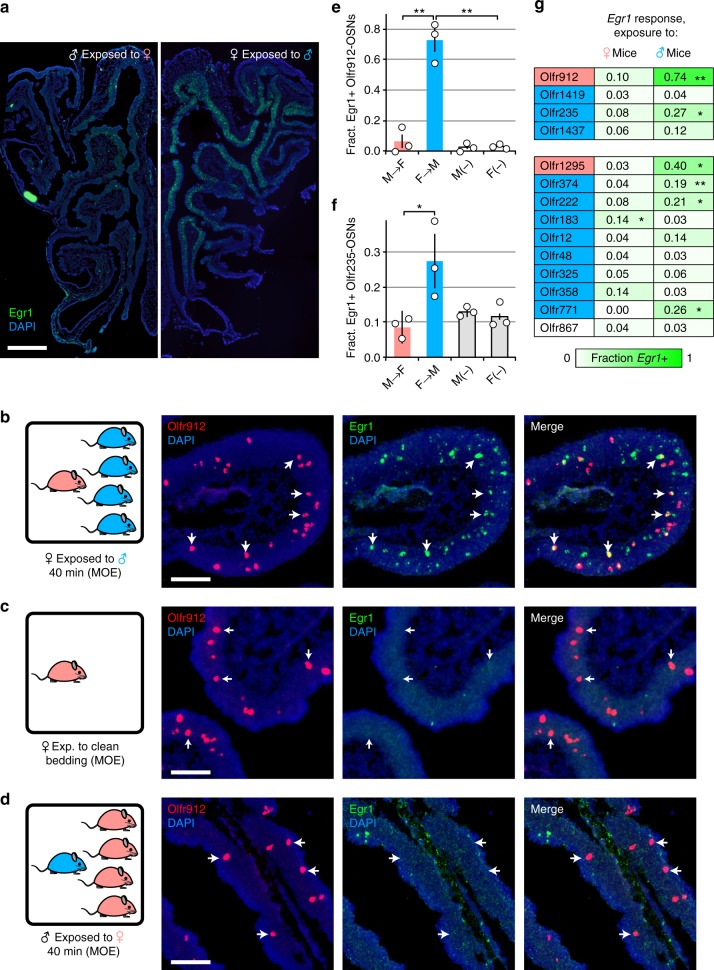
ORs differentially expressed between SF and SM mice detect sex-specific odors. **a** FISH analyses of *Egr1* expression within the MOE following exposure of a male mouse to a group of female mice (♂ exposed to ♀; left) or a female mouse to a group of male mice (♀ exposed to ♂; right). **b**–**d** Representative images of two-color RNA-FISH analyses of *Olfr912* and *Egr1* co-expression following exposure of a female mouse to a group of male mice (**b**), a female mouse to clean bedding (**c**), or a male mouse exposed to a group of female mice (**d**). Arrows: locations of representative *Olfr912*-expressing cells. **e**, **f** Quantification, based on two-color RNA-FISH, of the fraction of *Olfr912*-expressing (**e**) and *Olfr235*-expressing (**f**) OSNs that co-express *Egr1* within the MOE of a male mouse exposed to 4 female mice for 40 min (M→F), a female mouse exposed to 4 male mice for 40 min (F→M), a male mouse exposed to clean bedding (M(–)), or a female mouse exposed to clean bedding (F(–)).*p < 0.05, **p < 0.01 (two-tailed *t* test); *n* = 3 MOE sections (average of 52 *Olfr912*-expressing and 56 *Olfr235*-expressing OSNs/section). Error bars: s.e.m. Dots represent average values for individual sections. **g** Quantification, based on two-color RNA-FISH, of the fraction of the indicated OR-expressing OSNs that co-express *Egr1* within the MOE of individual male mice exposed to 4 females (♀ mice) or female mice exposed to 4 males (♂ mice) for 40 min SF-biased (SF > SM) and SM-biased (SM > SF) ORs (based on RNA-seq) are highlighted in pink and blue, respectively. ORs shown to have differential expression frequencies in SF and SM mice are grouped separately (*top*). *Olfr867* is a representative negative control OR. Statistical analysis results in **g** are based on comparisons between the fractions of OSNs co-expressing *Egr1* after exposure to either female or male mice. *p < 0.05, **p < 0.01 (two-tailed *t* test); *n* = 3 MOE sections (average of 60 specific OR-expressing OSNs/section). Scale bars for **a**: 500 µm; **b**–**d**: 100 µm

The observation that sex-specific differences in OR expression depend on whether mice experience a sex-separated or sex-combined environment suggests that differentially expressed ORs may detect odors that are emitted predominantly (or exclusively) by one sex. To test this hypothesis, we employed an immediate-early gene (IEG) assay to investigate whether ORs that are expressed differentially between SF and SM mice respond to sex-specific odors. IEGs are used extensively to identify neurons that are activated by particular stimuli^48^, and the IEGs *Fos* and *Egr1* have been used as markers of both OSN^49–51^ and VSN^25,35,39^ activation. Because IEGs may exhibit differences in their regulation by different classes of stimuli^48^, we used quantitative PCR (qPCR) to test a series of IEGs for their responsiveness to a group of male mice or to clean bedding within the MOE of female mice. Female mice exposed to male mice for 40 min showed significantly higher levels of *Fos*, *Rgs2*, and *Egr1* expression compared to clean bedding (Supplementary Fig. 5). FISH analyses further showed that *Fos* and *Egr1* are highly expressed in subsets of OSNs within mice exposed to either male or female mice (not shown), with a greater number of responsive neurons observed following exposure to males (Fig. 4a). We chose *Egr1* for use in subsequent experiments due to its high signal-to-noise ratio and sensitivity to sex-specific odor exposure.

We analyzed the co-expression of *Egr1* following a 40-min exposure to male mice, female mice, or clean bedding, with each of 21 ORs: 2 ORs expressed more highly in SF compared to SM mice (*Olfr912* and *Olfr1295*), 11 ORs expressed more highly in SM compared to SF (*Olfr1419*, *Olfr1437*, *Olfr235*, *Olfr374*, *Olfr12*, *Olfr48*, *Olfr771*, *Olfr183*, *Olfr222*, *Olfr325*, and *Olfr358*), and 8 ORs not differentially expressed (*Olfr867*, *Olfr958*, *Olfr1195*, *Olfr16*, *Olfr653*, *Olfr733*, *Olfr727*, *and Olfr167*), which served as negative controls. For 7 of the 13 differentially expressed ORs, we observed significant differences in the fraction of OSNs co-expressing *Egr1* following exposure to female mice versus male mice, as well as following exposure to male mice versus clean bedding (Fig. 4b–g). The significantly lower proportion of differentially responsive ORs among the negative control group (0 of 8) compared to the differentially expressed group (7 of 13) (*p* = 0.002; binomial test) indicates that the responsive ORs in the latter group were not identified by chance but are truly responsive to sex-specific odors. Interestingly, both of the SF-biased ORs, *Olfr912* and *Olfr1295*, responded robustly to male mice but not to clean bedding or female mice (Fig. 4b–e; Supplementary Fig. 4c-e), indicating that they are activated by male odors. Moreover, our findings that the expression levels of *Olfr912* and *Olfr1295* are significantly reduced in mice exposed to male odors (SM, CF, CM) compared to mice unexposed to male odors (SF) indicate that male odors may reduce the number of OSNs expressing these ORs (Fig. 3a–d; Supplementary Fig. 4a, b). Five of the SM-biased ORs analyzed showed significant responses to male mice, while one showed significant responses to females. Notably, six of the SM-biased ORs did not exhibit a significant response in the *Egr1* assay. Although the reason for this result is unclear, one possible explanation is that the *Egr1* assay may not detect all OSN activity, consistent with findings from IEG assays in other systems^52^. This might occur, for example, if an odor only weakly stimulates OSNs or if the odor is not specific to one sex but rather is emitted by both sexes but in different amounts. Additional experimental approaches will be needed to test this possibility.

### Differentially expressed vomeronasal receptors (VRs) detect sexually dimorphic odors

**Fig. 5:**
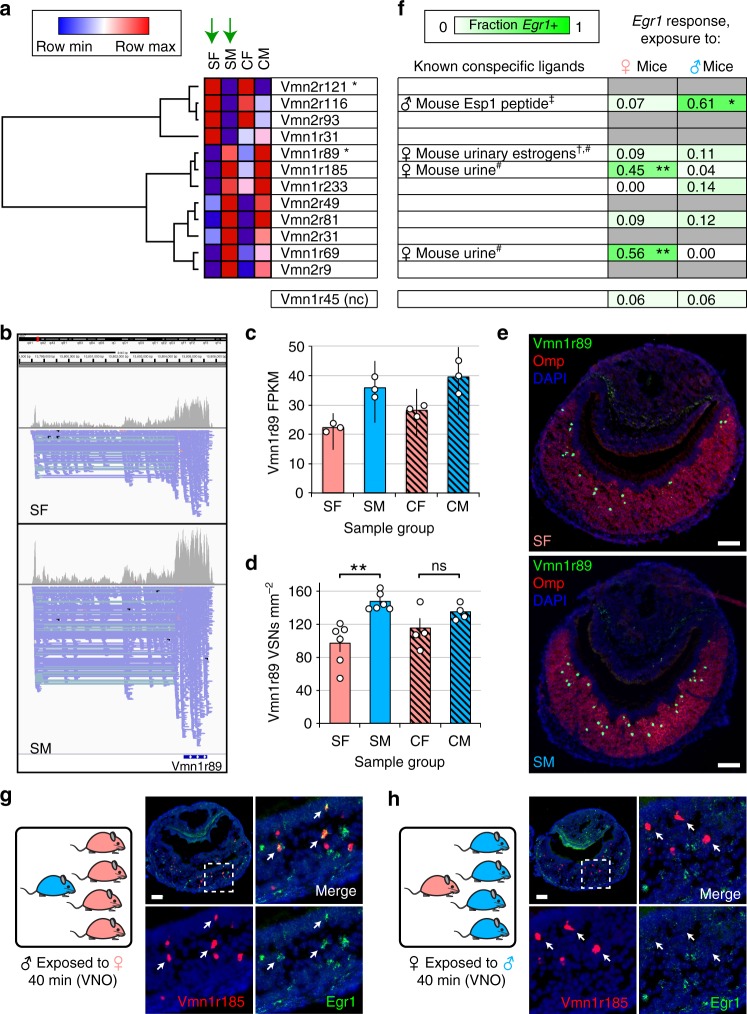
VRs with sex-specific expression differences exhibit sex-specific expression frequencies and detect sex-specific odors. **a** Hierarchical clustering of VRs identified via RNA-seq as differentially expressed between SF and SM mice (green arrows). VRs labeled asterisk, FDR < 0.05; other VRs shown, unadjusted *p* < 0.01 (Cufflinks output). **b** RNA-seq read alignments to *Vmn1r89* for SF and SM samples. Alignments from the three biological replicates in each experimental group were combined (SF, top; SM; bottom). Strand orientation: −, pink; +, blue. **c** VNO expression levels, based on RNA-seq, for *Vmn1r89*. Error bars: 95% c.i. **d** Quantification, using two-color RNA-FISH, of the frequency of *Vmn1r89*-expressing VSNs relative to all mature VSNs (based on *Omp* expression). Error bars: s.e.m. **p < 0.01 (two-tailed *t* test); *n* = 4–6 mice (5 sections/mouse; average of 17 *Vmn1r89*-expressing VSNs/section). Dots represent average values for individual mice. **e** Representative images of *Vmn1r89* expression in the VNO of the experimental mouse groups shown. **f** (Left) Known conspecific activators of the VRs shown in **a**, based on †: ref. ^39^, ‡: ref. ^35^, #: ref. ^38^. (Right) Quantification, based on two-color RNA-FISH, of the fraction of VSNs that co-express the VRs listed in **a** and *Egr1* within the VNOs of individual male mice exposed to 4 females (♀ mice) or females exposed to 4 males (♂ mice) for 40 min. *Vmn1r45* is a representative negative control VR. Statistical results in **f** are based on comparisons between the fractions of VSNs co-expressing *Egr1* after exposure to female or male mice. *p < 0.05, **p < 0.01 (two-tailed *t* test); *n* = 4 VNO sections (average of eight specific VR-expressing VSNs/section); gray fill: not tested. **g**, **h** Representative images of two-color RNA-FISH analyses of *Vmn1r185* co-expression with *Egr1* following exposure of a male mouse to four females (♂ exposed to ♀; g) or a female mouse to four males (♀ exposed to ♂; h). Arrows: locations of representative *Vmn1r185*-expressing cells. SF sex-separated females, SM sex-separated males, CF sex-combined females, SM sex-combined males. Scale bars: 100 µm

In contrast to the MOE, in which we observed little overlap in the identities of genes differentially expressed between SF and SM mice and those differentially expressed between CF and CM, a more substantial level of overlap was observed in the VNO between the same comparisons (Supplementary Fig 6a; Supplementary Data 9). As in the MOE, we found that a subset of the genes differentially expressed in the VNO between SF and SM mice are chemoreceptors: VRs belonging to both the type-1 (Vmn1r) and type-2 (Vmn2r) classes. Unlike ORs, however, we found that nearly as many VRs were differentially expressed between CF and CM mice (10) as between SF and SM (12) (Fig. 5a; Supplementary Fig. 6b, c). Interestingly, the majority of VRs differentially expressed in both comparisons were found to be identical (Fig. 5a; Supplementary Fig. 6c, e). These results indicate that, in the VNO, differences in the expression of VRs and other genes between male and female mice occur via mechanisms that are largely independent from exposure to opposite-sex odors and may instead be driven primarily by self-derived odors.

To examine whether VR expression differences observed by RNA-seq reflect differences in expression frequency, we again used RNA-FISH. Of the six VRs analyzed, two (*Vmn1r89* and *Vmn2r116*) exhibited expression frequencies closely paralleling transcript levels determined by RNA-seq, including significant differences between SF and SM mice (Fig. 5b–e; Supplementary Figs. 6f, 7a–d). Three additional VRs analyzed (*Vmn1r233*, *Vmn2r81*, and *Vmn1r69*) displayed expression frequency differences trending in the direction predicted by RNA-seq without reaching statistical significance (Supplementary Fig. 6f), while a fourth (*Vmn1r185*) showed negligible differences. These latter results may be explained, at least in part, by high levels of sequence homology between many VR genes, which can complicate FISH-based quantification of VR expression frequencies (Supplementary Table 1). Alternatively, or additionally, some VR expression differences observed by RNA-seq may reflect differences in cellular VR transcript levels. Taken together, these results indicate that a substantial fraction of the VR expression differences observed by RNA-seq between SF and SM mice reflect differences in the number of VSN subtypes in which the VRs are expressed.

Strikingly, several VRs that were differentially expressed between SF and SM mice by RNA-seq have been found previously to detect sex-specific odors^35,38,39^ (Fig. 5f, left). These include *Vmn2r116*, which is expressed more highly in female mice (Fig. 5a; Supplementary Fig. 7a–d) and is known to detect the male-specific exocrine gland peptide pheromone *Esp1*^25,35^, and *Vmn1r89*, *Vmn1r185*, and *Vmn1r69*, which are expressed more highly in male mice and are known to detect components of female urine^38^. Consistent with previous findings, we found via the *Egr1* assay that *Vmn2r116*-expressing VSNs respond specifically to male mice, while *Vmn1r185-* and *Vmn1r69*-expressing VSNs respond specifically to females (Fig. 5f–h; Supplementary Fig. 7e, f). The lack of significant *Egr1* co-expression observed in *Vmn1r89-*expressing VSNs, which reportedly detect urinary estrogens produced by female mice in estrus^38^, is also consistent with previous findings^39^ and might reflect the fact that the female mice used as stimuli were not in estrus. Likewise, it is possible that other differentially expressed VRs that did not exhibit responses to male or female odors detect odors that are emitted cyclically, are activated too weakly to trigger *Egr1* expression, or are activated by odors emitted in different quantities by both sexes. None of the six negative control VRs tested (*Vmn1r45*, *Vmn1r54*, *Vmn1r192*, *Vmn1r43*, *Vmn1r8*, and *Vmn1r215*) exhibited significantly differential responses to male-specific and female-specific odors in the *Egr1* assay. The significantly lower proportion of differentially responsive VRs among the negative control group (0 of 6) compared to the differentially expressed group (3 of 6) (*p* = 0.016; binomial test) provides further evidence that members of the latter group are truly responsive to sex-specific odors. Taken together, these results indicate that exposure to sex-specific odors can alter VR expression frequencies and that a prominent mechanism for such changes may be the shortening of the lifespan of specific VSN subtypes via chronic self-stimulation.

### Differentially expressed ORs are altered by naris occlusion

**Fig. 6:**
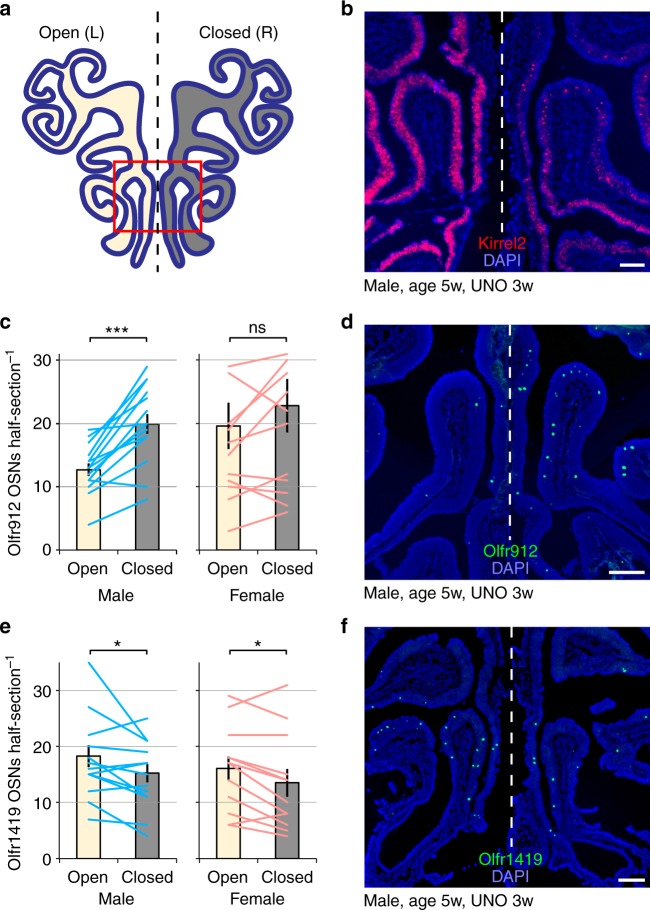
Olfactory deprivation via unilateral naris occlusion (UNO) alters the expression frequency of SF-biased and SM-biased ORs. **a** Schematic of a coronal MOE section following occlusion of the right nostril. The red box corresponds to the approximate region of the images shown in **b**, **d**, and **f**. **b** Confirmation of UNO efficiency via RNA-FISH analysis of the expression of *Kirrel2*^5,8^, an activity-dependent gene that is more strongly expressed on the open (left) side of the MOE. **c**, **e** Quantitation of *Olfr912*-expressing (**c**) and *Olfr1419*-expressing (**e**) OSNs on the open and closed sides of the MOEs of mice subjected to UNO. Each line represents a different MOE section. Error bars: s.e.m. *p < 0.05, ***p < 0.001 (two-tailed paired *t* test); *n* = 12 male and 16 female sections (taken from 4 mice of each sex). **d**, **f** Representative images of *Olfr912* (**d**) and *Olfr1419* (**f**) expression in the MOEs of mice subjected to occlusion of the right nostril. Scale bars: 200 µm

Although results from the *Egr1* assay suggest that the distinct olfactory experiences of sex-separated mice drive sex-specific differences in chemoreceptor expression, it is conceivable that these differences are instead caused by sex-specific hormonal differences between separated male and female mice. Thus, as a further test of the contribution of olfactory experience to the induction of sex-specific OR expression differences, we analyzed the expression frequencies of ORs following olfactory sensory deprivation via UNO, a procedure in which odorant-evoked activity on one side of the MOE is substantially reduced (Fig. 6a)^53^. We performed UNO on 2-week-old mice and analyzed OR expression after a period of 3 weeks of naris closure. We reasoned that, if the observed receptor changes are driven by olfactory experience, we might find altered expression frequencies of sex-biased ORs on the closed side of the MOE. After confirming UNO efficiency via RNA-FISH analysis of the expression of *Kirrel2*^5,8^, an activity-dependent gene that is expressed more strongly on the open side of the MOE (Fig. 6b), we quantified expression frequencies on the open and closed sides of the MOE for four ORs that are differentially expressed in SF and SM mice: *Olfr912*, *Olfr1419*, *Olfr1437*, and *Ofr235*, as well as a negative control OR (*Olfr867*) that is not differentially expressed between SF and SM mice. Three of the four SM-biased or SF-biased ORs exhibited significantly altered expression frequencies on the closed side of the MOE relative to the open side. While *Olfr912* was expressed more frequently on the closed side (Fig. 6c, d), *Olfr1419* (Fig. 6e, f) and *Olfr1437* (not shown) were expressed significantly more frequently on the open side. *Olfr235* and the negative control OR showed no significant difference in expression frequency on the open and closed sides (not shown). Interestingly, the effects on *Olfr912* expression were greater in males than in females, consistent with the hypothesis that *Olfr912* detects a male-biased odor that reduces OSN survival when presented chronically. In contrast, the effects on *Olfr1419* and *Olfr1437* were observed in both genders. The reason for the lack of gender specificity in the effects for *Olfr1419* and *Olfr1437* is unknown but might reflect the fact that mice of both sexes were present from the start of the UNO period until weaning. Nevertheless, these results support the hypothesis that olfactory experience contributes to the generation of sex-specific OR expression differences in sex-separated mice.

## Discussion

Results of the present study reveal that the separation of mice from members of the opposite sex for an extended period induces extensive differences in the olfactory sensory receptor repertoires of male and female mice. These results are consistent with previous findings that the olfactory environment can affect an individual’s OSN subtype composition^9–13^. Our results also indicate that these sex-separation-induced differences result, at least in part, from the distinct chemical profiles emitted by male and female mice^24–32^, which activate distinct subsets of OSNs^26^ and VSNs^21,24,27,32–39^. Thus, while male and female sex-separated mice receive olfactory stimulation only from members of the same sex (including themselves) and therefore have distinct olfactory experiences, male and female sex-combined mice receive olfactory stimulation from members of both sexes and therefore have similar olfactory experiences. Interestingly, we observed substantially fewer sex-specific gene expression differences in the VNO compared to the MOE in sex-separated mice. The reason for this disparity is unclear but may reflect a smaller capacity for plasticity in the VNO compared to the MOE.

It is notable that previous studies comparing gene expression in the MOE and VNO tissues of male and female mice have observed relatively few differences compared to sex-separated mice in the present study^45,54,55^. Two possible differences in study design may help to explain this discrepancy: First, mice analyzed in previous studies were younger (typically 4–10 weeks of age) than those of the present study (6 months of age), and thus spent a smaller proportion of their lives in a sex-separated environment. Since changes in OR/VR expression frequency appear to involve neuronal turnover and occur over the course of multiple weeks or even months^11,14^, time may be a critical factor in these changes. Second, mice used in previous studies may not have been housed under conditions that completely isolated them from opposite sex odors, which may be important.

**Fig. 7:**
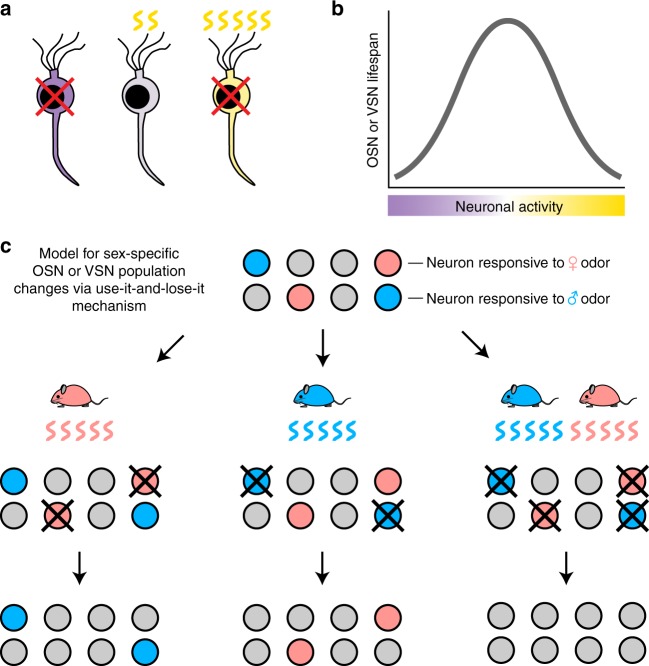
Model for how OSN and VSN subtypes become differentially abundant in SF and SM mice. **a** Very low (violet) or very high (yellow) levels of activity may shorten OSN/VSN lifespan, while a moderate (gray) level of activity may lengthen their lifespan. **b** An optimal range of neuronal activity may be required to maximize OSN/VSN lifespan. Distinct OSN/VSN subtypes may be affected differently by olfactory manipulation due to differences in their basal level of activity. **c** Schematic for how the distinct olfactory environments experienced by sex-separated females and males may induce differences in the abundance of OSN/VSN subtypes (for simplicity, only the use-it-and-lose-it mechanism is shown). Initially, an individual’s OSN/VSN populations contain neuron subtypes that are responsive to odors specific to female mice (pink circles), male mice (blue circles), or neither (gray). Upon sex separation of females (left) or males (middle), OSNs/VSNs that are responsive to female-specific or male-specific odors, respectively, are chronically stimulated and thus selectively depleted from the population. Under sex-combined conditions (right), OSNs/VSNs that are responsive to both female-specific and male-specific odors are chronically stimulated and depleted from the population

Our findings that sex separation alters the expression frequencies of specific ORs and VRs raises the question of how these changes occur. Previous studies have shown that olfactory deprivation^9–11,13^ and enrichment^14^ can cause both increases and decreases in the expression levels of distinct ORs, at least some of which reflect changes in the abundance of corresponding OSN subtypes within the MOE. Our findings indicate that a similar type of plasticity occurs within the VNO to enable changes in the relative representation of VRs. Although the precise mechanisms by which these changes occur remain to be determined, we postulate that they may be explained by two factors: (1) Neurons that receive activity within an optimal range may have an enhanced lifespan and become enriched within the population relative to those that do not (Fig. 7a, b). (2) Distinct OSN/VSN subtypes may be affected differently by deprivation of sex-specific odors via sex separation due to differences in their sex-combined level of activity, both ligand-independent^56^ and ligand-dependent. For example, sex separation may shorten the lifespan of neurons that are weakly stimulated under sex-combined conditions via a use-it-or-lose-it mechanism while the same condition may lengthen the lifespan of neurons that are strongly stimulated under sex-combined conditions via relief from a use-it-and-lose-it mechanism.

We found that sex separation induces differences in the expression of several ORs and VRs via a use-it-and-lose-it type mechanism (Fig. 7c). Among ORs, this mechanism is exemplified by *Olfr912*, which we have found is activated by male-specific odors (Fig. 4b–e). Under conditions in which male mice are present (SM, CF, CM cages), *Olfr912*-expressing OSNs are significantly reduced in abundance compared to conditions in which males are absent (SF cages; Fig. 3a–d). Moreover, olfactory deprivation via UNO significantly increased the abundance of *Olfr912*-expressing OSNs on the closed side in male but not in female mice (Fig. 6c, d). Among VRs, this mechanism is exemplified by *Vmn2r116*, *Vmn1r185*, and *Vmn1r69*. *Vmn2r116*-expressing VSNs, which are activated by exposure to the male exocrine gland-specific peptide ESP1^35^ as well as to male mice (Supplementary Fig. 7e, f), are significantly less abundant in SM compared to SF mice (Fig. 5a; Supplementary Figs. 6f, 7a–d). These results may explain why male mice of strains expressing high levels of ESP1 are unresponsive to the peptide, which was hypothesized to be caused by VSN desensitization due to the self-secretion of ESP1^35^. Likewise, *Vmn1r185-*expressing and *Vmn1r69-*expressing VSNs, which are activated by exposure to female-specific urinary components^38^ as well as to female mice (Fig. 5f–h), are significantly less abundant in SF compared to SM mice (Fig. 5a). These results may help explain the finding that a subset of VSNs that detect sulfated steroids can be silenced in male mice by long-term exposure to sulfated steroids or to female urine^21^.

Results from the present study also suggest a role for a mechanism in which odors increase the representation of specific ORs/VRs, perhaps via a use-it-or-lose-it mechanism (Fig. 7a). Such a mechanism may be exemplified by *Olfr1419*, an OR that is more frequently expressed in mice exposed to male odors (SM, CF, CM mice) than in mice unexposed to male odors (SF mice) (Fig. 2d; Suppl Fig. 3a–d) and is decreased on the closed side of the MOE following olfactory deprivation via UNO (Fig. 6e, f).

Might sex-specific differences in hormone levels (e.g., as a result of mating, birth, pup removal, or same sex co-housing) play a causal role in sex-specific gene expression differences? Indeed, recent studies have shown that hormones can act on both OSNs^57^ and VSNs^23^ to change their response properties but whether these effects can alter the expression frequency of specific ORs or VRs is unknown. If such changes were to occur, they would be expected to occur predominantly under sex-combined conditions due to the fluctuating sex-specific hormones related to mating and reproduction in these mice^58,59^. In fact, however, our findings indicate that sex-specific differences are significantly more numerous in sex-separated mice, suggesting that sex-specific hormone effects related to reproduction do not play a major role in the majority of sex-specific differences observed in this study. Nevertheless, our findings do not rule out a role for hormones in mediating sex-specific receptor expression differences observed specifically in sex-combined mice, such as for *Olfr1280*, *Olfr53*, *Vmn1r73*, and *Vmn2r74* (Supplementary Figs. 2e, 6c). Nor do our findings rule out the possibility that same-sex housing causes sex-specific hormone differences that contribute to sex-specific OR/VR frequency differences. Indeed, although the results of our activity assay and olfactory deprivation experiments strongly indicate a role for olfactory experience in the induction of sex-specific chemoreceptor expression differences, it is conceivable that hormones also play a contributing role.

Plasticity within the OSN population has been postulated to enable adaptation of an individual’s olfactory system for the sensitive detection of salient odors, which may vary from one environment to another^11^. Opposite-sex odors are highly salient, but the extent to which animals are exposed to these odors in nature may vary substantially among individuals. Thus the changes identified here may be physiologically relevant to animals in nature. We speculate that mechanisms that alter an individual’s OSN and VSN populations may play a role in adjusting their sensitivity to sex-specific odors. This hypothesis is supported by findings that the long-term exposure of male mice to a sulfated steroid in female urine reduces both VSN responses to the ligand as well as male attraction to female urine^21^. Results presented here indicate that the reduced VSN responsiveness observed in that study may be due to the depletion of specific VSN subtype(s) and that similar changes may occur broadly in both the VNO and MOE. Moreover, the depletion of specific OSN/VSN subtypes due to chronic odor exposure could conceivably play an adaptive role by reducing olfactory signaling in animals that emit the odors as well as in other animals that are in frequent contact. Conversely, odors that trigger an enrichment of specific OSN/VSN subtypes could also play an adaptive role by enhancing the sensitivity of individuals to sex-specific odors that may be present in low concentrations or emitted only occasionally. Considering the diversity of changes observed within olfactory sensory organs as a function of exposure to members of the opposite sex, the effects of these changes on behavior are expected to be complex. Moreover, distinguishing the physiological effects of changes that occur within the sensory organs from those known to occur elsewhere in the nervous system^60^ is a challenging but important future objective.

The identification of receptors that detect gender-specific odors is a long-standing objective in olfactory research due to its potential to facilitate investigations of conspecific communication and sex-specific behaviors in mammals^40,61^. We have found evidence that several of the ORs and VRs identified in this study detect sex-specific odors and anticipate that these findings will provide new opportunities for future investigations. The identification of ORs that detect sex-specific cues may prove especially valuable, as receptors that detect sex-specific odors in the MOE have heretofore remained largely unidentified.

## Methods

### Experimental animals

All procedures involving animals were carried out in accordance with NIH standards and approved by the University of Wyoming and Harvard University Institutional Animal Care and Use Committees (IACUC). A total of 70 C57Bl/6 mice (equal mix of male and female, 5 weeks–6 months of age) were used to obtain the tissue samples described here, including 36 mice for RNA-seq analyses (see Supplementary Fig. 1), 24 mice for OR/VR expression frequency analyses, and 10 mice for *Egr1* and OR/VR colocalization analyses following odor exposure.

### Preparation of tissues from sex-separated and sex-combined mice

C57Bl/6 mice were subjected to either sex-separated (SF and SM samples) or sex-combined (CF and CM samples) conditions, in which animals were housed four females per cage (SF), four males per cage (SM), or two females and two males per cage (CF and CM) from weaning (PD 21) until 6 months of age (Supplementary Fig. 1). Sex-separated and sex-combined cages were set up by transferring mice from their birth cages at age PD 21 into cages containing littermates or, when necessary, age-matched mice from different litters. At the time of transfer, PD 21 mice were randomly assigned to one of the four experimental groups. All mice in a given cage were added to experimental cages and removed for euthanasia on the same dates as their cage-mates in order to maximize consistency with respect to the developmental status and experiences of mice within each sex/condition group (SF, SM, CF, CM). At the time of weaning, SF and SM cages were transferred to rooms containing only mice of the same sex to avoid exposure to opposite-sex odors from cages in the same room. Pups born in the sex-combined cages were euthanized within 1 day of birth to minimize exposure to pup odors. Mice were monitored both visually and via auditory cues for signs of aggression, typically 3–4 times/day, with no evidence of fighting observed in any of the mouse groups. These observations are consistent with previous findings that multiple male C57Bl/6 mice can cohabitate from the time of weaning indefinitely into adulthood without fighting^42^. At 6 months of age, mice were sacrificed and MOE, VNO, and OB tissues were dissected. Tissues from one half the mice were frozen immediately and stored at −80 °C until use for RNA-seq. Tissues from the other half were placed in a cryomold containing Optimal Cutting Temperature (OCT), flash-frozen, and stored at −80 °C until sectioned. Tissue blocks were cut into 12-μm-thick cryo-sections, placed onto slides, and stored at −80 °C until use in FISH analysis.

### Preparation of tissues for IEG analysis

Experimental female and male C57Bl/6 mice were housed under sex-separated conditions until 8–10 weeks of age. At the time of odor exposure, mice were removed individually from the sex-separated conditions and placed in a cage containing four mice of the opposite sex for 40 min Experimental mice were then removed from the odor exposure cage and immediately sacrificed. MOE and VNO tissues were then removed, placed in a cryomold containing OCT, flash-frozen, and stored at −80 °C until sectioned. Tissue blocks were cut into 12-μm-thick cryo-sections, placed onto slides, and stored at −80 °C until use in FISH analysis.

### Unilateral naris occlusion

Fourteen-day-old mice were anesthetized using isoflurane (completeness of anesthesia confirmed through a tail pinch) and then immediately subjected to electrocautery for ~5 s on the right nostril under a dissecting microscope, with care taken to avoid contact of the electrocautery unit with any non-superficial tissues. Mice were examined on a daily basis to ensure complete blockage of the right nostril through scar formation (typically ~3–5 days after the procedure) and normal mouse development and activity. Mice were sacrificed at 5 weeks of age and MOE tissues were dissected and frozen in OCT.

### RNA-seq analysis

For each combination of tissue (MOE, VNO, OB), sex (female, male), and condition (sex-separated, sex-combined), six individual RNA samples were prepared via mechanical homogenization in Trizol Reagent (Life Technologies) following the manufacturer’s protocol, resulting in a total of 108 samples (Supplementary Fig. 1). Trizol-purified RNA samples were quantified using a NanoDrop instrument (ThermoFisher Scientific). To generate the RNA samples used to prepare 36 the RNA-seq libraries used in this study, we pooled equal quantities of three RNA samples extracted from tissues dissected from three individual non-sibling mice from three different cages. The pooling of samples from different litters/cages was done to minimize the possibility of any cage-specific or litter-specific effects on experience and gene expression. Pooled RNA samples were further purified using an RNeasy Plus Mini Kit (Qiagen) to generate 36 samples, representing 3 biological replicates per combination of tissue/sex/condition. Integrity of the RNA was analyzed using a 2100 Bioanalyzer (Agilent). All MOE, VNO, and OB samples had RNA integrity number values of at least 8.3, 7.3, and 8.8, respectively.

Using the TruSeq Stranded Total RNA with Ribo-Zero Gold Kit (Illumina), each RNA sample was depleted of ribosomal RNA and used to prepare an RNA-seq library tagged with a unique barcode. Library identity and quality were confirmed via qPCR analysis using primers specific for genes expressed in the MOE (*Cnga2*, *Hist2h2be*), VNO (*Vmn1r51*, *Hist2h2be*), and male tissues (*Utyl*). Libraries were quantified using a Qubit instrument (ThermoFisher Scientific). Libraries were paired-end sequenced (2×50 bases) to a depth of ~40 million reads/sample (~20 million fragments/ replicate) using a HighSeq 2000 instrument (Illumina). RNA-seq data were analyzed using the Galaxy platform (https://usegalaxy.org)^62^. For each sample, sequence pairs were aligned to the genome using Tophat2^63,64^, resulting in high-quality alignment of ~80% of the read pairs. The RNA samples and RNA-seq datasets used in this study have been characterized in a separate paper^43^. Gene-lavel and transcript-level analyses of differential gene and transcript expression were performed using Cufflinks^64^. Significance testing for differential expression was performed on all genes with a minimum alignment count of 10 fragments. In MOE, VNO, and OB tissues, 14,735, 14,691, and 13,548 genes, respectively, fit this criterion, which was also used to define the number of expressed genes in each tissue. Venn diagrams were generated using Bio Venn (http://www.biovenn.nl/index.php)^65^.

### GO and hierarchical clustering analyses

GO analysis was performed using the GOrilla software (http://cbl-gorilla.cs.technion.ac.il/)^66^, using the single ranked list of genes mode. Reported enrichment *p* values are FDR-adjusted using the Benjamini–Hochberg method^67^. Hierarchical clustering analysis was performed using GenePattern (https://genepattern.broadinstitute.org)^68^.

### In situ hybridization (ISH) probe design and production

ISH probes were designed to span 500–1000 base pairs and were targeted to CDS, untranslated region, and intron gene regions (Supplementary Table 1). Probes were designed to minimize cross-hybridization with off-target mRNAs, which was assessed using BLAST. For the detection of specific ORs and VRs, probes targeting multiple gene regions were typically generated and tested. In some cases, the probes affording the best signal-to-noise ratio were anticipated to have the potential to detect off-target mRNAs (Supplementary Table 1). Probe sequences were amplified by PCR using specific primers (Supplementary Table 1), inserted into the pCRII-TOPO vector (Invitrogen), and confirmed by restriction analysis and sequencing. Digoxigenin (DIG)-labeled and 2,4-dinitrophenyl (DNP)-labeled antisense RNA probes were generated from 1 μg of linearized plasmid template using T7 or Sp6 polymerases (Promega) and DIG-11-UTP (Roche) or DNP-11-UTP (Perkin Elmer), treated with DNaseI (Promega), ethanol precipitated, and dissolved in a 30-μL volume of water.

### One-color RNA-FISH

Slide-mounted sections were warmed (37 °C, 10 min), equilibrated in phosphate-buffered saline (PBS; pH 7.2; 5 min, room temperature [RT]), fixed in paraformaldehyde (PFA; 4% in PBS; 10 min, RT), washed in PBS (3 min, RT), permeabilized with Triton-X-100 (0.5% in PBS; 10 min, RT) followed by sodium dodecyl sulfate (1% in PBS; 3 min, RT), washed in PBS (3× 3 min, RT), incubated in acetylation solution (triethanolamine [0.1 M; pH 7.5], acetic anhydride [0.25%]; 10 min, RT), washed in PBS (3× 3 min, RT), incubated in hybridization solution (formamide [50%], SSC [5×], Denhardts [5×], yeast tRNA [250 μg/mL], herring sperm DNA [500 μg/mL], heparin [50 μg/mL], EDTA [2.5 mM], Tween-20 [0.1%], CHAPS [0.25%]; 30 min, RT), hybridized with a DIG-labeled antisense RNA probe (1:750 in hybridization solution; 16 h, 65 °C), washed with SSC (2×; 1× 5 min, 65 °C), washed with SSC (0.2×; 4× 20 min, 65 °C), incubated in H_2_O_2_ (3% in TN [Tris-HCl (0.1 M; pH 7.5), 0.15 M NaCl]; 30 min, RT), washed in TNT (Tween-20 [0.05%] in TN; 5× 3 min, RT), incubated in 1,3,5-trinitrobenzene (TNB; Blocking Reagent [Perkin Elmer; 0.05% in TN]; 30 min, RT), incubated with anti-DIG-POD antibody (Roche; 1:1000 in TNB; 12 h, 4 °C), and washed in TNT (3× 20 min, RT). Fluorescent signals were generated using the Tyramide Signal Amplification (TSA) Plus Fluorescein Kit (Perkin Elmer) according to the manufacturer’s instructions. Slides were washed in TNT (2× 3 min, RT), incubated in 4,6-diamidino-2-phenylindole (DAPI) (300 nM in TN; 3 min, RT), washed in TNT (1× 3 min, RT), and mounted using Vectashield (Vector Laboratories).

### Two-color RNA-FISH

Two-color RNA-FISH was performed as described for one-color RNA-FISH, with the following modifications: Tissue sections were simultaneously hybridized with both DIG-labeled and DNP-labeled antisense RNA probes (1:1000 each in hybridization solution). To avoid inadvertent cross-hybridization of vector sequences contained within antisense RNA probes used on the same slide, care was taken to generate both probes using clones in which the gene fragments were inserted into the vector in the same orientation. Following incubation in TNB (30 min, RT), sections were incubated with anti-DNP-horseradish peroxidase antibody (Perkin Elmer; 1:500 in TNB; 3 h at 25 °C) and washed in TNT (3× 20 min, RT). Fluorescent signals corresponding to the DNP-labeled probes were generated using the TSA Plus Fluorescein Kit, after which sections were washed in TNT (2× 3 min, RT), incubated in H_2_O_2_ (3% in TN; 1 h, RT), washed in TNT (5× 3 min, RT), incubated with anti-DIG-POD antibody (1:1000 in TNB; 12 h, 4 °C), and washed in TNT (3× 20 min, RT). Fluorescent signals corresponding to the DIG-labeled probe were generated using the TSA Plus Cyanine5 Kit (Perkin Elmer) according to the manufacturer’s instructions. Slides were washed in TNT (2× 3 min, RT), incubated in DAPI (300 nM in TN; 3 min, RT), washed in TNT (1× 3 min, RT), and mounted using Vectashield.

### Image acquisition and processing

Images were acquired using an Axio Imager M2 microscope with an automated stage and Zen Blue software (Zeiss). Mosaic images were stitched and each fluorescence channel was adjusted individually to enhance contrast and reduce background using the Zen Blue software. Images were rotated and cropped using Adobe Photoshop and labeled using Adobe Illustrator (Adobe Systems).

### Quantification of OR and VR expression frequencies

Fluorescent OSN and VSN counts corresponding to an individual mouse were determined from a series of 5–6 stained coronal sections located ~400 μm apart and spanning the anterior–posterior length of the MOE or VNO tissue, respectively. Fluorescent cell counting was performed manually with the experimenter blinded to the sample group. OR and VR expression frequencies per unit of epithelial area were calculated based on areas of OMP expression, which were determined using the Zen Blue software (Zeiss).

### qPCR analysis of IEG expression

cDNA samples for qPCR analysis were prepared using the QuantiTect Reverse Transcription Kit (Qiagen) starting from whole MOE RNA prepared using Trizol Reagent and purified using an RNeasy Miniprep Kit. qPCR experiments were performed using the QuantiTect SYBR Green PCR Kit (Qiagen) with an MJ Opticon 2 instrument (Bio-Rad). Primer pairs (Integrated DNA Technologies) were designed using the Primer-BLAST tool (NCBI). Primer efficiencies were assessed using standard curves and pairs exhibiting efficiencies of >99% were used for analysis.

### Statistics

For RNA-seq analyses, we used a sample size of *n* = 3 biological replicates (each generated from RNA pooled from 3 individual mice) and a sequencing depth of ~20 million fragments/sample, consistent with the recommended guidelines for differential gene expression analysis^69^. Gene-level and transcript-level analyses of differential gene expression were performed using Cufflinks^64^, with significance testing performed on all genes/transcripts with a minimum alignment count of 10 fragments. We used an FDR of <0.05 as a significance threshold for identification of overall gene expression differences, including those used in GO analyses (Fig. 2c). Significance testing for differences between the proportion of genes differentially expressed between two different comparisons was performed using the N-1 Chi-squared test^70^. For analyses involving ORs and VRs alone, we used a significance threshold of *p* < 0.01 (unadjusted), which enabled consideration of ORs/VRs that do not meet the *q* < 0.05 significance threshold due to low transcript abundance but may nevertheless exhibit differential expression. In support of this approach, our RNA-FISH analyses confirmed that ORs (e.g., *Olfr235*) and VRs (e.g., *Vmn2r116*) that met the *p* < 0.01 but not the *q* < 0.05 threshold have significantly different expression frequencies in SF and SM mice (Fig. 3e–h; Supplementary Figs. 2f, 5f, and 7a–d). Receptors have been marked according to the significance threshold that they meet for differential expression by RNA-seq (Figs. 2d, 5a; Supplementary Figs. 2c–e, 6c, d). For comparisons of OSN/VSN frequencies between different mice and of fractions of OSNs/VSNs that co-express *Egr1*, we used a two-tailed unpaired *t* test. We used the binomial test to determine whether there are significant differences between the proportions of differentially expressed and control receptors that respond to sex-specific odors in the *Egr1* assay. For comparisons of OSN counts on the two sides of MOE sections from UNO-treated mice, we used a two-tailed paired *t* test. For all OSN/VSN comparisons, we used a significance threshold of *p* < 0.05. As a pre-established criterion, sections that were not 90% intact were excluded from the analysis. To ensure adequate power to detect significance, we based the sample size on the number of expected OR-expressing and VR-expressing cells per mouse or section. We found that sample sizes of *n* = 4 mice (5 sections/mouse) for OR frequencies, 6 mice (5 sections/mouse) for VR frequencies, 3 sections for fractions of *Egr1*-co-expressing cells, and 12 sections for OSN counts in UNO sections were adequate to identify significant differences. For qPCR analysis, we used a two-tailed unpaired *t* test with a significance threshold of *p* < 0.05; a sample size of *n* = 2 was sufficient to detect significant differences in this assay.

## Electronic supplementary material

Supplementary Information

Description of Additional Supplementary Files

Supplementary Data 1

Supplementary Data 2

Supplementary Data 3

Supplementary Data 4

Supplementary Data 5

Supplementary Data 6

Supplementary Data 7

Supplementary Data 8

Supplementary Data 9

Peer Review File

## Data Availability

RNA-seq data files in FASTQ format were deposited at NCBI Sequence Read Archive, accession SRP136494 (2018), and are available for download. This archive contains a total of 144 FASTQ files resulting from paired-end sequencing for each of the 36 samples on two lanes. FPKM values for each sample were deposited at NCBI Gene Expression Omnibus, accession GSE112352 (2018), and are available for download. The RNA-seq datasets used here have been further described and characterized in a separate paper^43^.
