# Supplementary material for: Sex separation induces differences in the olfactory sensory receptor repertoires of male and female mice
Source: Nat Commun. 2018 Dec 4;9:5081. doi: 10.1038/s41467-018-07120-1 (PMC6279840; doi:10.1038/s41467-018-07120-1)
Supplement: Supplementary file 1 — Supplementary Information [file 41467_2018_7120_MOESM1_ESM.pdf]

Supplementary Information for:

Sex separation induces differences in the olfactory sensory receptor repertoires of male and female mice

Carl van der Linden, Susanne Jakob, Pooja Gupta, Catherine Dulac, Stephen W. Santoro

Supplementary Figures

Supplementary Figure 1. Experimental design and samples used.

Supplementary Figure 2. Analysis of OR genes that are differentially expressed in the MOE between different experimental groups.

Supplementary Figure 3. Differences in OR expression between SF and SM mice reflect differences in the abundance of corresponding OSN subtypes, as shown for two additional ORs.

Supplementary Figure 4. *Olfr1295* is expressed in a female-biased manner in sex-separated mice and responds to male-specific odors.

Supplementary Figure 5. Quantitative PCR (qPCR) analysis of the expression of immediate-early genes (IEGs) in the MOEs of female mice exposed to either groups of male mice (♀ exposed to ♂) or to clean bedding (♀ clean bedding).

Supplementary Figure 6. Analysis of genes, including VRs, that are differentially expressed in the VNO between different experimental groups.

Supplementary Figure 7. *Vmn2r116* is expressed in a female-biased manner in both sex-separated and -combined mice and responds to male-specific odors.

Supplementary Table

Supplementary Table 1. *In situ* hybridization probe design

Supplementary Figure 1

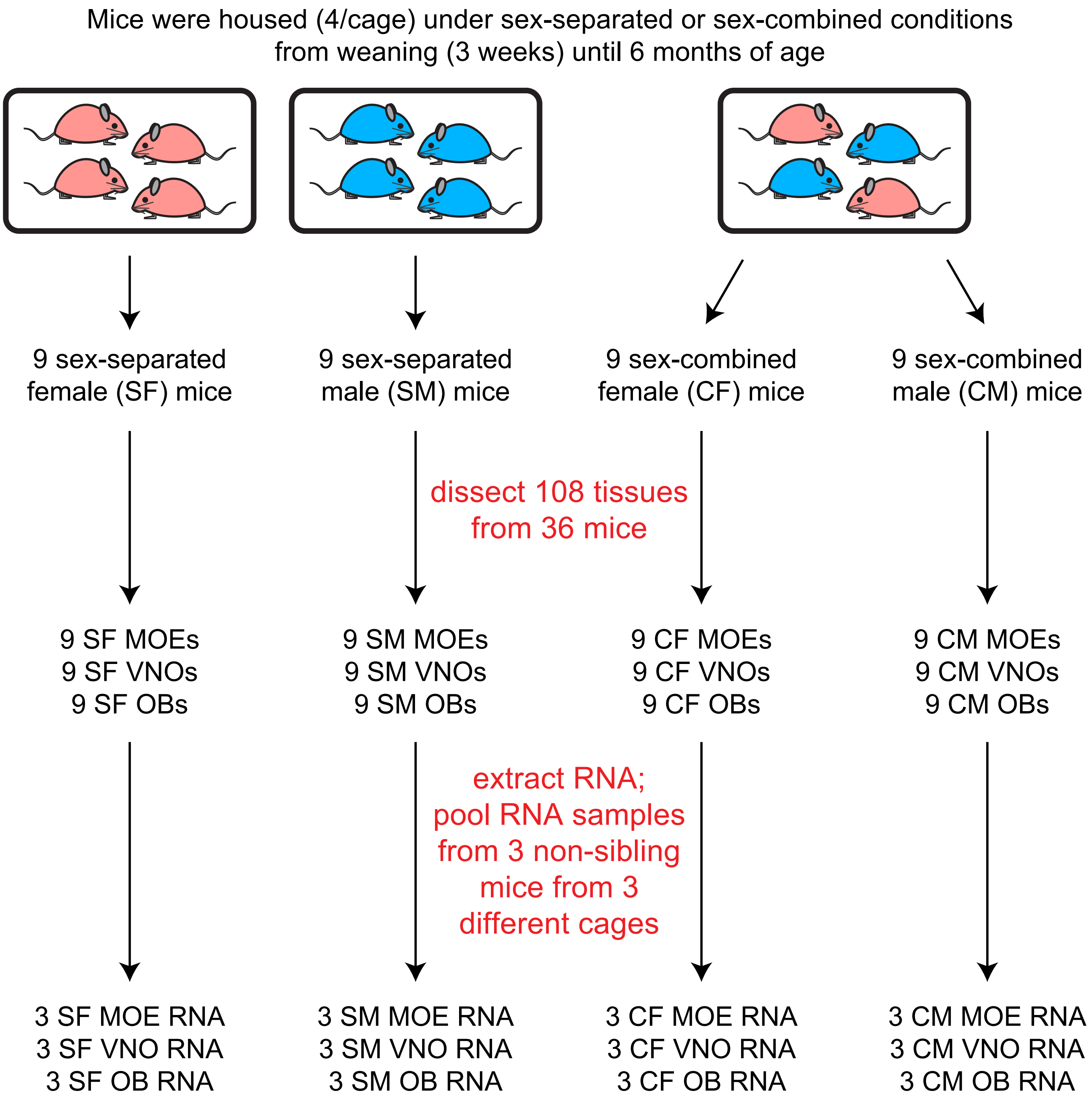

Supplementary Figure 1. Experimental design and samples used. From weaning (P21) until 6 months of age, mice experienced either a sex-separated environment, in which they were housed either 4 females/cage (SF mice; *left*) or 4 males/cage (SM mice; *middle*), or a sex-combined environment (CF and CM mice; *right*), in which they were housed 2 females + 2 males/cage. MOE, VNO, and OB tissues were dissected from each of 9 mice per sex/condition combination, resulting in a total of 108 tissue samples. RNA was extracted from each tissue sample and pooled in groups of 3, resulting in 36 RNA samples (3 biological replicates per sex/condition/tissue combination), and used to generate RNA-seq libraries.

Supplementary Figure 2

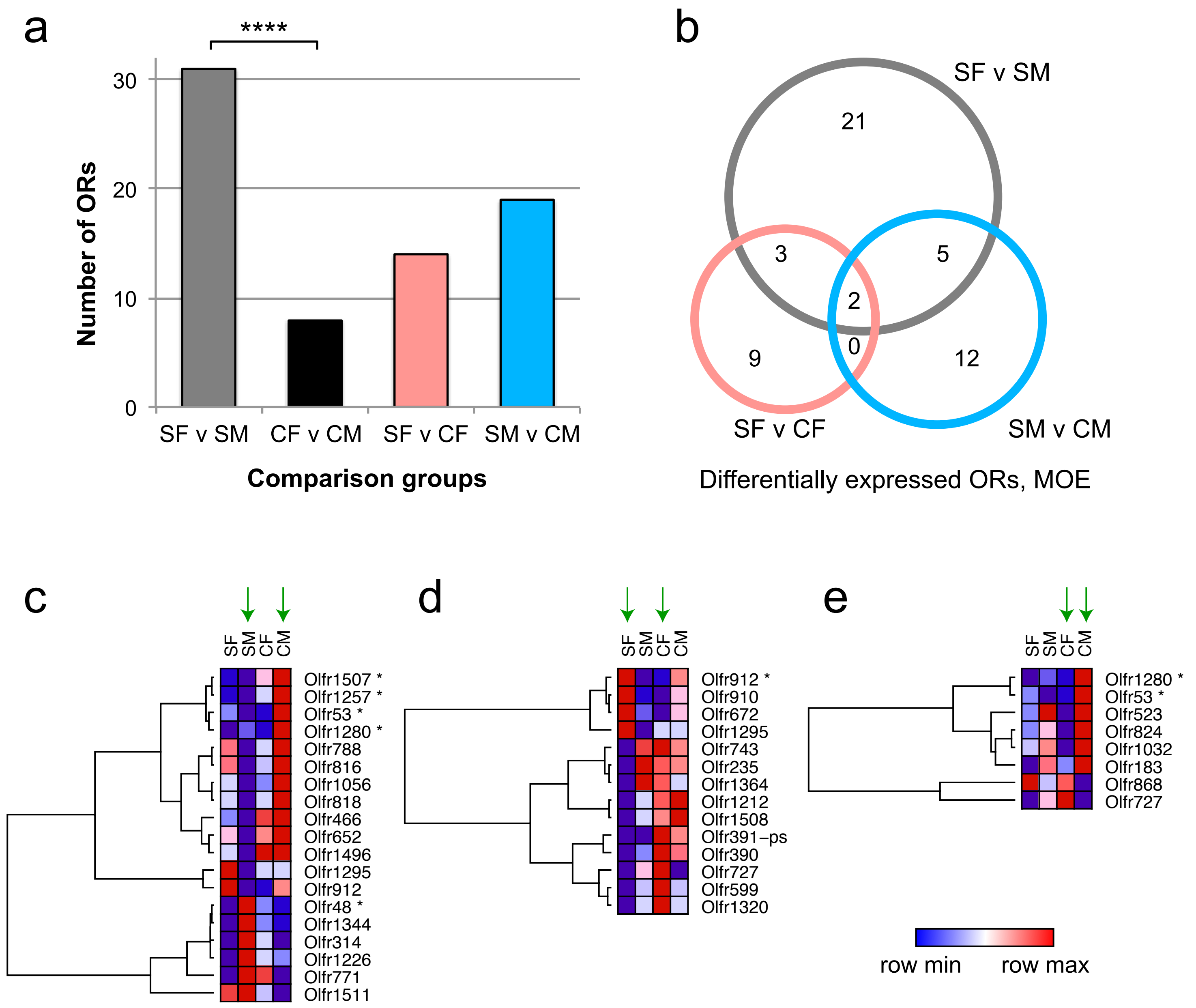

f

OR	Frequency SF SM^{-1}	P-value SF v SM 4 mice	(20 sections)	Frequency CF CM^{-1}	P-value CF v CM 4 mice	(20 sections)
Olfr141	0.93	0.38	(0.73)			
Olfr912	2.09	0.050	(0.0016)	0.84	0.61	(0.030)
Olfr1295	1.08	0.70	(0.61)			
Olfr976	0.86	0.12	(0.48)			
Olfr1419	0.48	0.11	(0.0045)	0.90	0.83	(0.64)
Olfr235	0.52	0.00095	(0.0000037)	1.27	0.085	(0.080)
Olfr48	0.75	0.33	(0.21)			
Olfr1437	0.51	0.140	(0.0033)	1.32	0.069	(0.052)

Supplementary Figure 2. Analysis of OR genes that are differentially expressed in the MOE between different experimental groups. (**a**) Number of OR genes that were identified *via* RNA-seq as differentially expressed (unadjusted $p < 0.01$) in the MOE between the indicated experimental groups. (**b**) Venn diagrams of OR genes identified *via* RNA-seq as differentially expressed (unadjusted $p < 0.01$) between the indicated experimental groups. (**c**–**e**) Hierarchical clustering of OR genes identified *via* RNA-seq as differentially expressed between SM and CM mice (*green arrows*; c), between SF and CF mice (*green arrows*; d), and between CF and CM mice (*green arrows*; e). ORs labeled *, FDR < 0.05; other ORs shown, unadjusted $p < 0.01$. (**f**) Quantification, by two-color RNA FISH, of the relative expression frequencies of representative OR-encoding genes that were identified *via* RNA-seq as differentially expressed (unadjusted $p < 0.01$) between SF and SM mice. OR expression frequencies for each experimental group are based on counting of specific OR-expressing cells relative to the area of all mature OSNs (detected by *Omp*). p-values for differential expression between the experimental groups shown were calculated using a two-tailed t-test based on mice: $n = 4$ (5 sections/mouse; average of 28 OSNs/section) or MOE sections (*parentheses*): $n = 20$ (average of 28 OSNs/section).

Supplementary Figure 3

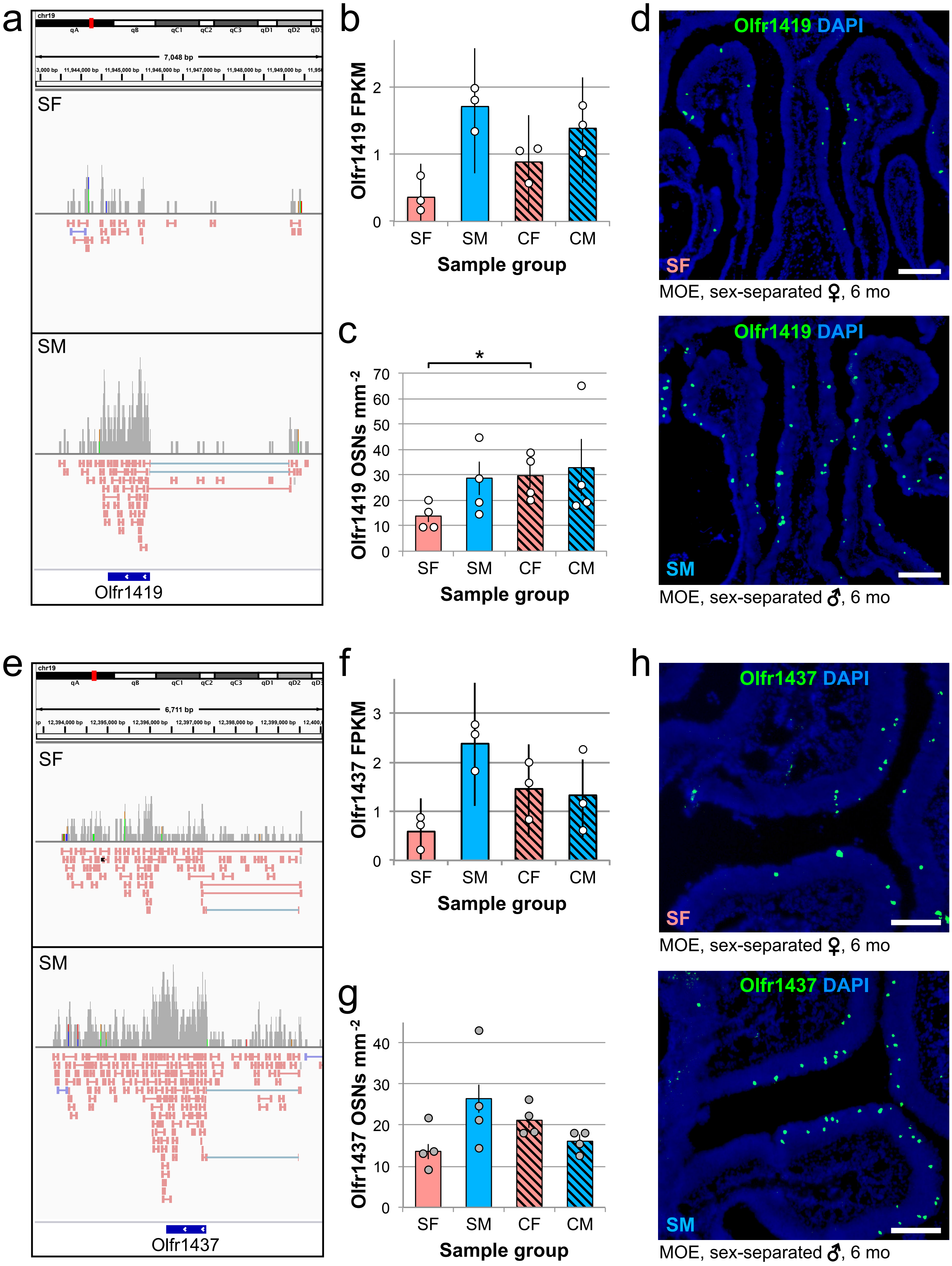

Supplementary Figure 3. Differences in OR expression between SF and SM mice reflect differences in the abundance of corresponding OSN subtypes, as shown for two additional ORs. (**a**, **e**) RNA-seq read alignments to *Olfr1419* (a) and *Olfr1437* (e) for SF and SM samples. For simplicity, alignments from the three biological replicates in each experimental group were combined (SF, *top*; SM; *bottom*). Strand orientation: - strand, *pink*; + strand, *blue*. (**b**, **f**) Expression levels, determined by RNA-seq FPKM values for *Olfr1419* (b) and *Olfr1437* (f) in the MOEs of the experimental mouse groups shown. Error bars: 95% c.i.. (**c**, **g**) Quantification, using two-color RNA-FISH, of the frequency of *Olfr1419*-expressing (c) and *Olfr1437*-expressing (g) OSNs relative to the area of all mature OSNs (based on *Omp* expression) for each experimental group. Error bars: s.e.m. *$p < 0.05$ (two-tailed t-test); n = 4 mice (5 sections/mouse; average of 21 *Olfr1419*-expressing and 17 *Olfr1437*-expressing OSNs/section). Dots represent average values for individual mice. (**d**, **h**) Representative images of *Olfr1419* (d) and *Olfr1437* (h) expression in the MOEs of the experimental mouse groups shown. Scale bars: 200 µm.

Supplementary Figure 4

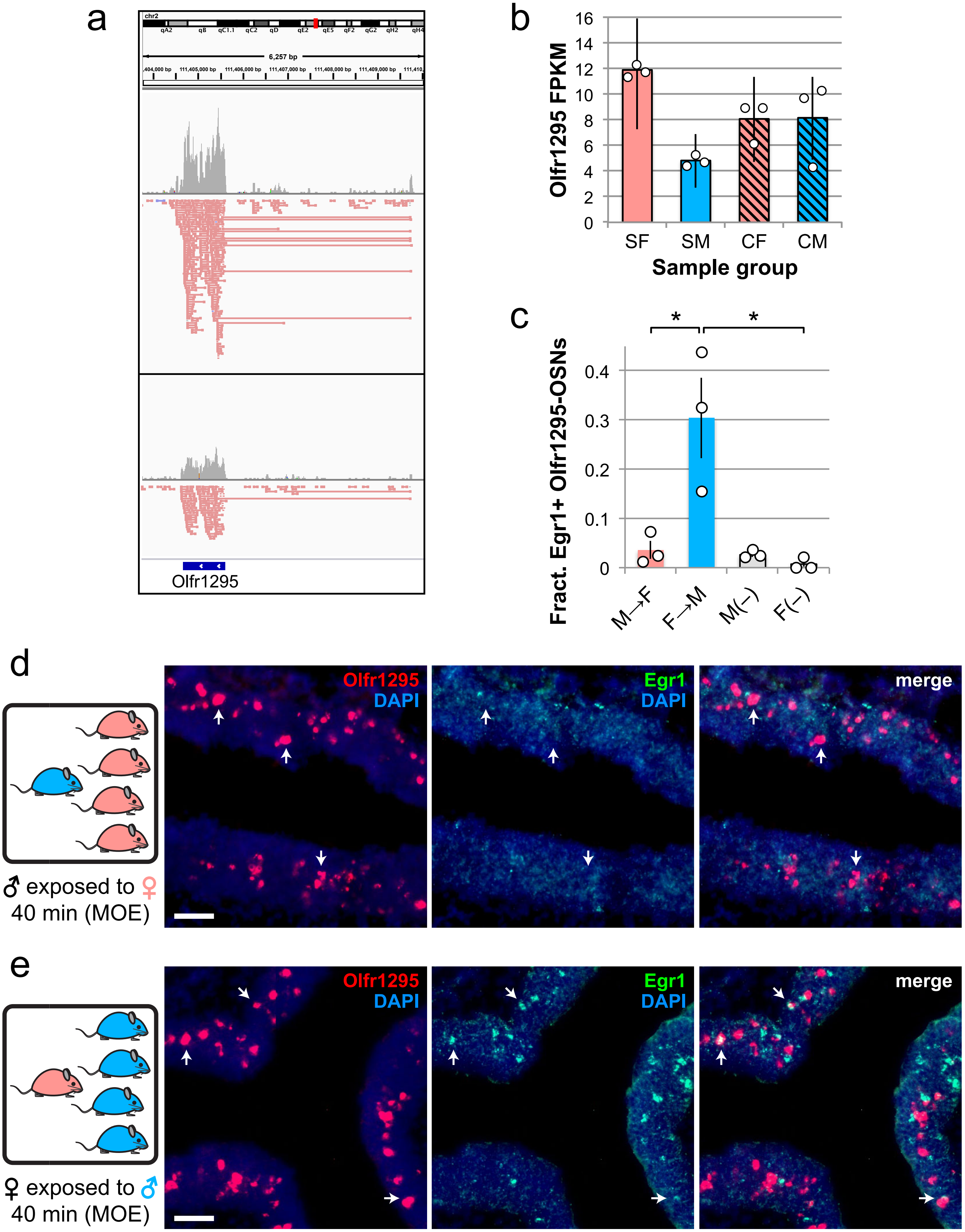

Supplementary Figure 4. *Olfr1295* is expressed in a female-biased manner in sex-separated mice and responds to male-specific odors. (**a**) RNA-seq read alignments to *Olfr1295* for SF and SM samples. For simplicity, alignments from the three biological replicates in each experimental group were combined (SF, *top*; SM; *bottom*). Strand orientation is indicated by color: - strand, *pink*; + strand, *blue*. (**b**) Expression levels, determined by RNA-seq FPKM values for *Olfr1295* in the MOEs of the experimental mouse groups shown. Error bars: 95% c.i. (**c**) Quantification, based on two-color RNA FISH, of the fraction of *Olfr1295*-expressing OSNs that co-express *Egr1* within the MOE of a male mouse exposed to 4 female mice for 40 min. (M→F), a female mouse exposed to 4 male mice for 40 min. (F→M), a male mouse exposed to clean bedding (M(-)), or a female mouse exposed to clean bedding (F(–)). *$p < 0.05$ (two-tailed t-test); $n = 3$ MOE sections (average of 73 *Olfr1295*-expressing OSNs/section). Error bars: s.e.m. Dots represent average values for individual sections. (**d**, **e**) Representative images of two-color RNA FISH analyses of *Olfr1295* co-expression with *Egr1* following exposure of a female mouse to a group of male mice (♂ exposed to ♀; d) or a male mouse to a group of female mice (♀ exposed to ♂; e). *Arrows*: locations of representative *Olfr1295*-expressing cells. Scale bars: 50 µm.

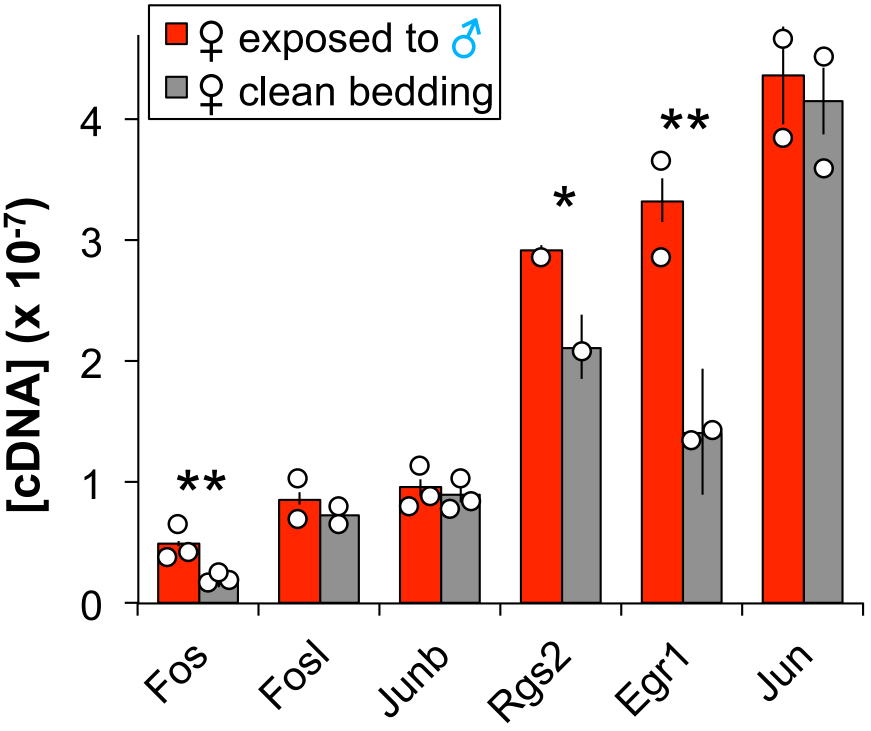

Supplementary Figure 5. Quantitative PCR (qPCR) analysis of the expression of immediate-early genes (IEGs) in the MOEs of female mice exposed to either groups of male mice (♀ exposed to ♂) or to clean bedding (♀ clean bedding). *$p < 0.05$, **$p < 0.01$ (two-tailed t-test; n = 2 mice). Error bars: s.d.

Supplementary Figure 6

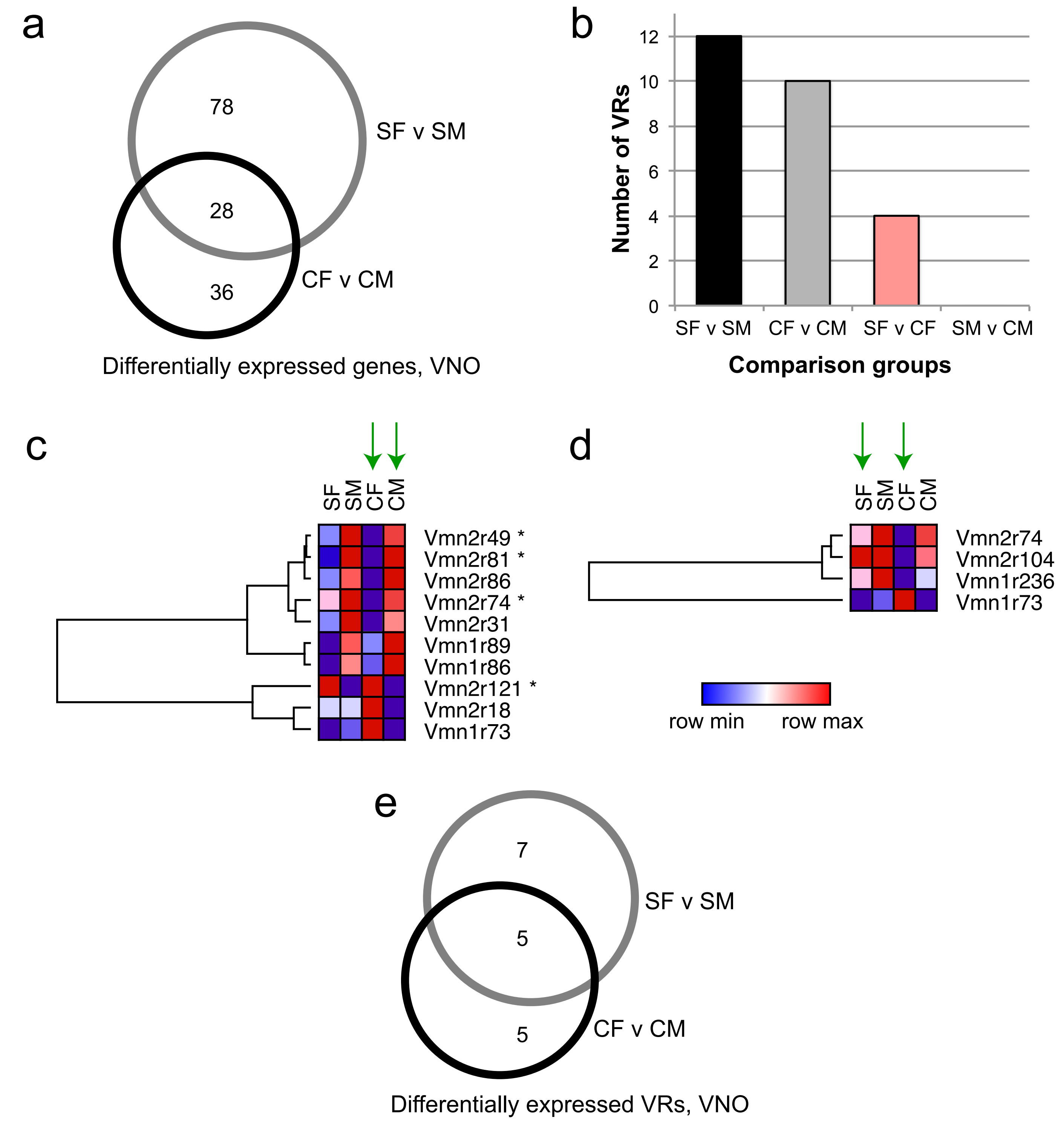

f

VR	Count ratio SF SM^{-1}	P-value SF v SM 6 mice (30 sections)	Count ratio CF CM^{-1}	P-value CF v CM 6 mice (30 sections)
Vmn2r116	1.40	0.050 (0.025)	1.06	0.78 (0.70)
Vmn1r89	0.66	0.0013 (0.0000091)	0.85	0.16 (0.073)
Vmn1r185	1.03	0.83 (0.74)		
Vmn1r233	0.87	0.33 (0.20)		
Vmn2r81	0.91	0.75 (0.54)		
Vmn1r69	0.90	0.37 (0.42)		

Supplementary Figure 6. Analysis of genes, including VRs, that are differentially expressed in the VNO between different experimental groups. (**a**) Venn diagram of overlap of genes identified *via* RNA-seq as significantly differentially expressed (FDR < 0.05) between male and female sex-separated (SF vs. SM) and sex combined (CF vs. CM) mice. (**b**) Number of VR-encoding genes that were identified *via* RNA-seq as differentially expressed (unadjusted $p < 0.01$) in the VNO between the indicated experimental groups. (**c**, **d**) Hierarchical clustering of VR-encoding genes identified *via* RNA-seq as differentially expressed between SF and SM mice (*green arrows*; c) and between SF and CF mice (*green arrows*; d). VRs labeled *, FDR < 0.05; other ORs shown, unadjusted $p < 0.01$. (**e**) Venn diagram of VR-encoding genes identified *via* RNA-seq as differentially expressed (unadjusted $p < 0.01$) between male and female sex-separated (SF vs. SM) and sex combined (CF vs. CM) mice. (**f**) Quantification, using two-color RNA FISH, of the relative expression frequencies of representative VRs that were identified *via* RNA-seq as differentially expressed (unadjusted $p < 0.01$) between SF and SM mice. VR expression frequencies for each experimental group are based on counting of specific VR-expressing cells relative to the area of all mature VSNs (detected by *Omp*). p-values for differential expression between the experimental mouse groups shown were calculated using two-tailed t-tests based on mice: $n = 6$ (5 sections/mouse; average of 9 VSNs/section), or VNO sections (*parentheses*): $n = 30$ (average of 9 VSNs/section).

Supplementary Figure 7

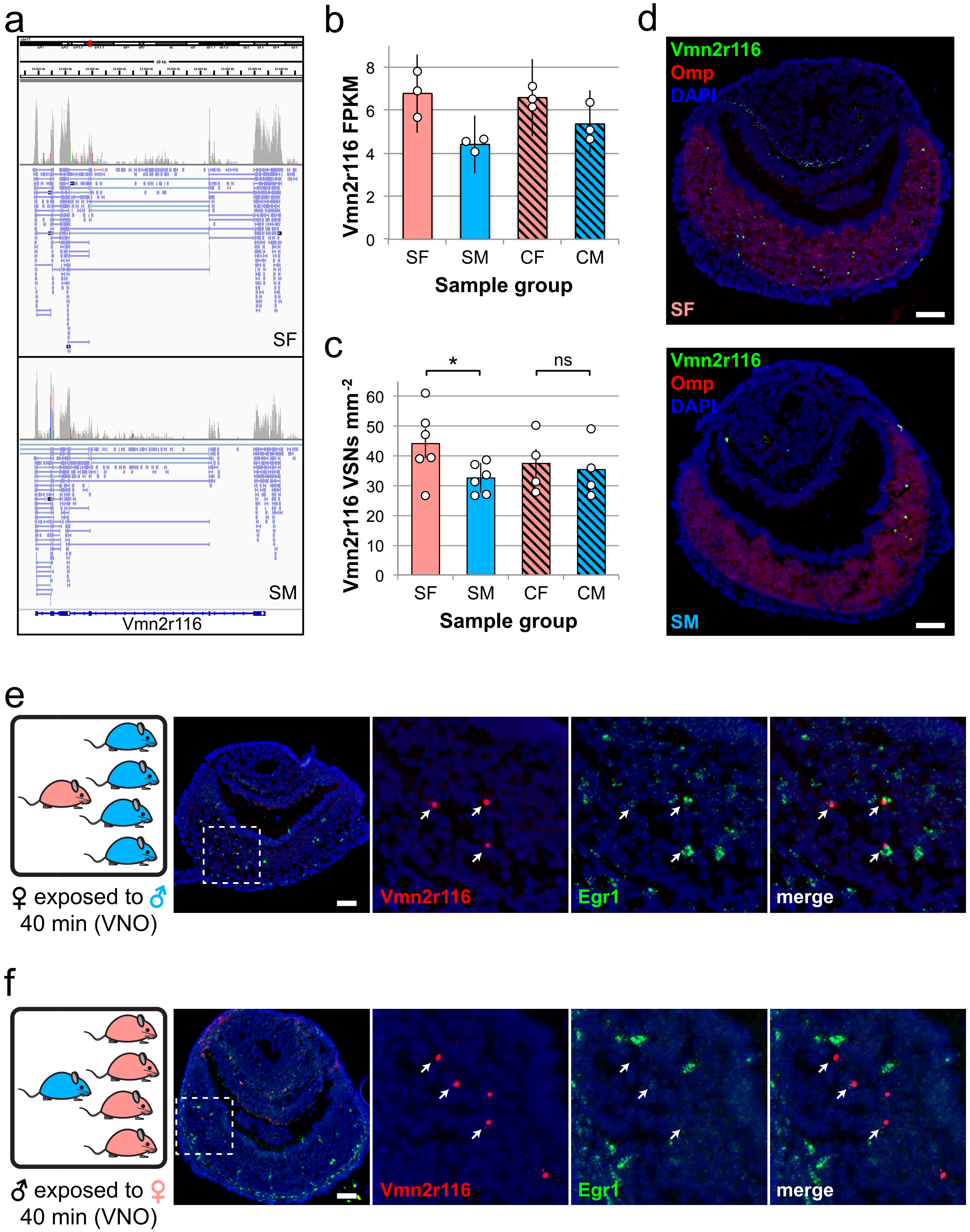

Supplementary Figure 7. *Vmn2r116* is expressed in a female-biased manner in both sex-separated and -combined mice and responds to male-specific odors. (**a**) RNA-seq read alignments to *Vmn2r116* for SF and SM samples. For simplicity, alignments from the three biological replicates in each experimental group were combined (SF, *top*; SM; *bottom*). Strand orientation is indicated by color: - strand, *pink*; + strand, *blue*. (**b**) Expression levels, determined by RNA-seq FPKM values for *Vmn2r116* in the VNOs of the experimental mouse groups shown. Error bars: 95% c.i. (**c**) Quantification, using two-color RNA-FISH, of the frequency of *Vmn2r116*-expressing VSNs relative to all mature VSNs (based on *Omp* expression). $^{**}p < 0.01$ (two-tailed *t*-test); $n = 4 – 6$ mice (5 sections/mouse; average of 6 *Vmn2r116*-expressing VSNs/section). Error bars: s.e.m. Dots represent average values for individual mice. (**d**) Representative images of *Vmn2r116* expression in the VNOs of the experimental mouse groups shown (SF, *top*; SM; *bottom*). (**e**, **f**) Representative images of two-color RNA FISH analyses of *Vmn2r116* co-expression with *Egr1* following exposure of a female mouse to a group of male mice (♀ exposed to ♂; e) or a male mouse to a group of female mice (♂ exposed to ♀; f). *Arrows*: locations of representative *Vmn2r116*-expressing cells. Scale bars: 100 µm.

Supplementary Table 1. *In situ* hybridization probe design

Olfr141	OSN counting	3' of CDS	+ 1182	ACGTTGGCTTTG CATGTGTTC	AAAGTCCCAGTC TCTGACTCC	992	1	0	1095
Olfr912	OSN counting, Egr1 assay	CDS/3' of CDS	+ 483	TGCATCCTGAGA CTGACTTTCT	GCTGTGTCAAAT GCAAGATTTTAAA	500	46	3	44, 145,146, 147, 229, 874, 875, 878, 883, 884, 885, 887, 888, 889, 890, 901, 902, 906, 907, 908, 910, 914, 915, 916, 917, 918, 920, 921, 922, 923, 933, 935, 936, 937, 943, 952, 954, 955, 957, 967, 968, 969, 970, 971, 983
Olfr976	OSN counting	CDS/3' of CDS	+ 949	AAGCAATGCAAC CTTTGGGG	TCAGGTTCTTCCA GGAGGCA	705	0	0	
Olfr1419	OSN counting, Egr1 assay	CDS	+ 33	TTCCACTTCCGC CCCTTTTC	ACTTCTTCTGCGA CATGCCT	753	0	0	
Olfr1437	OSN counting, Egr1 assay	3' of CDS	+ 1190	TTCAGCCCTTGG TCTTGTCC	CCTGACGGTGAG AAGTGTCC	753	1	0	1436
Olfr235	OSN counting, Egr1 assay	5' of CDS	- 7217	TCAACAGCATGT TCAGGGGA	GCCTTTTTCTCAC CTGGGCT	673	2	0	1431, 1433,
Olfr48	OSN counting, Egr1 assay	CDS/3' of CDS	+ 691	CCCTCTCCACCT GTGTCTCT	TGGAGGCACTGT CTGTTTCA	904	3	0	1258, 1254, 1240
Olfr374	Egr1 assay	CDS	+ 37	TGCTGCTAGGCT TGTCACAG	ACTGCTGTGAAG ATGCGTGT	637	31	6	8, 18, 39, 54, 57, 191, 351, 353, 365, 371, 412, 527, 531, 707, 708, 741, 742, 845, 853, 860, 870, 871, 1351, 1352, 1353, 1354, 1356, 1375, 1377, 1378, 1402
Olfr12	Egr1 assay	5' of CDS	- 1992	ACTGGAGGCTAC ACATCCCT	GGTGTGGTTAGAT GCAGCCT	502	0	0	
Olfr771	Egr1 assay	CDS	+ 245	AGCAACCGGAG ACAGGTCAA	GGTTCAACATGG GAGCAATCG	605	26	0	777, 800, 770, 780, 781, 772, 773, 799, 769, 791, 794, 768, 821, 792, 814, 815, 798, 774, 805, 776, 805, 810, 816, 775, 802, 820
Olfr183	Egr1 assay	CDS	+ 252	TGCAGGGTGCA CATACATGA	CTCTTGGACAAG GGCAAGGT	537	13	4	196, 186, 187, 197, 193, 191, 190, 192, 194, 203, 205, 198, 209
Olfr222	Egr1 assay	5' of CDS/CDS	- 342	AGCCAGTGGAA AATGGCGTA	ACTGAACGTGAAT GACGCCT	674	8	0	317, 1360, 1359, 1367, 138, 136, 124, 15
Olfr325	Egr1 assay	CDS/3' of CDS	+ 853	TCACCCCTCTGT TGAACCCA	TAGGGAAAACCA CGGACCAT	522	0	0	
Olfr358	Egr1 assay	CDS	+ 248	ACACACACTCTC TGTGGTTCG	GATGGCTTGAGG TACATGCG	537	2	1	360, 361
Vmn2r116	VSN counting, Egr1 assay	Intron	+ 3212	AGACACAGAGA GAGATGGGTTA	TATGTTTGTGCTT GATAATGTCTTAG A	582	6	11	2r112, 2r111, 2r115, 2r117, 2r114, 2r113
Vmn2r81	VSN counting, Egr1 assay	3' of CDS	+ 46247	ACAAATTTCACA CACTATGACTGC	TGCTCCAAAAATC AGTGCCC	577	2	0	2r82, 2r80
Vmn1r233	VSN counting, Egr1 assay	5' of CDS	- 1709	TCCATATGACCT TTCTGGGGC	GTTTCACAAGGG CCCCTGAT	402	0	0	
Vmn1r185	VSN counting, Egr1 assay	5' of CDS/CDS	- 103	TGAAGCTCCATG GTTAATGGC	CACAATGGCTGTT CCATTTCCC	518	7	4	1r184, 1r71, 1r69, 1r67, 1r68, 1r225, 1r228
Vmn1r69	VSN counting, Egr1 assay	5' of CDS	- 1354	TATGGGCCCCTT TCTGGTTC	AGTGATCTGCATG GATGGGC	531	2	0	1r68, 1r67
Vmn1r89	VSN counting, Egr1 assay	5' of CDS	- 6123	GCAGCCTTTCCA AGACACTTTGT	ACTACTGTTTTCT GCAGGATAGTGG	828	2	0	1r87, 1r88
