# Supplementary material for: Sex separation induces differences in the olfactory sensory receptor repertoires of male and female mice
Source: Nat Commun. 2018 Dec 4;9:5081. doi: 10.1038/s41467-018-07120-1 (PMC6279840; doi:10.1038/s41467-018-07120-1)
Supplement: Supplementary file 3 — Supplementary Data 1 [file 41467_2018_7120_MOESM3_ESM.pdf]

Description of additional supplementary files for:

Sex separation induces differences in the olfactory sensory receptor repertoires of male and female mice

Carl van der Linden, Susanne Jakob, Pooja Gupta, Catherine Dulac, Stephen W. Santoro

File Name: Supplementary Data 1
Description: MOE sample differential gene expression

File Name: Supplementary Data 2
Description: VNO sample differential gene expression

File Name: Supplementary Data 3
Description: MOE sample differential gene expression

File Name: Supplementary Data 4
Description: Common genes differentially expressed between SF and SM mice within MOE, VNO, and OB tissues

File Name: Supplementary Data 5
Description: Common genes differentially expressed between CF and CM mice within MOE, VNO, and OB tissues

File Name: Supplementary Data 6
Description: Common genes differentially expressed within the MOE between SF and SM mice, SF and CF mice, and SM and CM mice

File Name: Supplementary Data 7
Description: Common genes differentially expressed within the VNO between SF and SM mice, SF and CF mice, and SM and CM mice

File Name: Supplementary Data 8
Description: Common genes differentially expressed within the MOE between SF and SM mice and CF and CM mice

File Name: Supplementary Data 9
Description: Common genes differentially expressed within the VNO between SF and SM mice and CF and CM mice
