# Supplementary material for: Sex separation induces differences in the olfactory sensory receptor repertoires of male and female mice
Source: Nat Commun. 2018 Dec 4;9:5081. doi: 10.1038/s41467-018-07120-1 (PMC6279840; doi:10.1038/s41467-018-07120-1)
Supplement: Supplementary file 2 — Description of Additional Supplementary Files [file 41467_2018_7120_MOESM2_ESM.pdf]

Reviewers' Comments:

Reviewer #1:
Remarks to the Author:
Linden et al tested the effects of the social context (being in the presence of same sex only conspecifics or mixed gender) on the cellular and molecular properties of the mouse olfactory system (from weaning until 6 months of age). Therefore, they had sex-separated females (SF), sex-separated males (SM) and sex-combined males and females (CF and CM). They analyzed the MOE, VNO and OB of the manipulated animals by RNAseq and histology (including using IEGs to test the responsiveness of OSN/VSN whose OR/VRs were differentially expressed).

The authors found more differentially expressed genes between SF and SM than between CF and CM. Chemoreceptors made up a large percentage of the differentially expressed genes and the differential expression was correlated with the number of sensory neurons expressing the receptor. Finally, they also show that many of the differentially expressed receptors detect male or female specific odors. These results were previously shown in the MOS, but the authors extend it to the vomeronasal system.

The authors claim that the differences they find in the olfactory system are due to olfactory scene created by the sex separation and that these differences probably arise by the loss of specific SN (when they are stimulated by specific odors in the combined group).

The study is a small tour de force and seems well done to me (as far as I understand, there were 6 samples for each condition and in the SF/SM group these come from 3 independent cages and in the CF/CM they come from 6 independent cages; it would be nice to have the full experimental design in the supplementary information, including the origin of the animals: are there siblings between animals distributed through the different cages?). Founder effects could interfere with the expression of chemoreceptors.

However, similar studies have been done previously, in particular I would like to emphasize the study by Ibarra-Soria et al, eLife 2017. Very similar results were observed for the MOE. In this paper the manipulations were different (embryos were transferred at very early stage between mothers, animals were cross-fostered or exposed to artificial odorants), but the observed phenomena was the same: changes in the expression of chemoreceptors and the correlated abundance of OSN expressing the receptor. In the current study, the authors claim to perform a manipulation where the animals were exposed to a different socially driven olfactory experience. It is true that they find differences in the composition/organization of the olfactory system. However, I do not think they can argue about what drives the observed changes.

The 4 experimental groups of this manuscript cannot be compared. SF housed for 6 months will get along well. In contrast, SM housed for 6 months will fight and establish hierarchy (I am actually surprised they could maintain 4 males in a cage for 6 months in a healthy condition; even if the males are sexually naïve, in my lab we start observing aggressive encounters post puberty and males must be separated to prevent barbering, biting and other aggressions). Finally, in the CF/CM group, animals had sex, females went through pregnancy an delivery several times. The neuroendocrine state of these animals is completely different and therefore I do not think we can pinpoint any cause to the observed results. For example, Shingo et al, (Shingo et al 2003. Pregnancy-stimulated neurogenesis in the adult female forebrain mediated by prolactin. Science) showed that neurogenesis changes through pregnancy and even mating. The odor context is different indeed, but the behaviour of the animals is even less comparable. Also, for example, the stress level of some of the SM in the sex-separated group must have been quite high. Even though the influence of stress hormones on the OE

is controversial, it seems like chronic exposure to cortisol has an effect of the OB according to Siopi et al, J Neuro 2016. The same can be extended to sex hormones, which are not comparable across the different groups.

So, my point is the following: if you want to just claim that animals that experience different social environment have different olfactory system, I think that is reasonable. But that has been shown before several times. In contrast, if the goal was to pinpoint the differences to the olfactory environment, I do not think it is possible with the experimental design used in this study. There are so many different variables that are not comparable across the groups that it is impossible in my view to do it. I acknowledge that perfect experiment (having sex-separated and sex-combined mice cohabiting and performing the same behaviour is literally impossible). But in this case, maybe a simpler exposure to bedding (or even full animals separated by a perforated wall where mice could exchange volatile and non-volatile cues) would make it possible to attribute the changes to the olfactory scene.

My main concerns are therefore with the main claim of the ms. I do not think the authors can claim that "Sex separation induced differences that appear to result from the fact that male and female mice emit distinct chemical profiles" even if some of the differences are observed in OR/VR that specifically respond to male/females cues. Despite the IEGs results, the authors do not test the biological relevance of the differential expression: for example, do SF have higher discriminability power than CF? The authors could test the sex discrimination capacities of SF versus CF.

Reviewer #2:
Remarks to the Author:
The manuscript by Van der Linden et al. examines the long-term effects of environment (housed by sex or co-housed) on gene expression changes in mammalian olfactory systems. The authors use a combination of RNA-seq and RNA-FISH on olfactory tissues, finding evidence for sex- and experience-dependent changes in gene expression. The authors provide detailed analysis of specific genes, focusing on chemosensory receptors in the main olfactory and vomeronasal epithelia. Two-color RNA-FISH was used to investigate odor sensitivities of differentially expressed chemosensory receptors, finding that some differentially expressed genes appear to be enriched following environmental exposure to cognate odors, whereas other differentially expressed genes appear to be diminished in the presence of cognate odors. In general, the data are clearly presented, but several concerns about analysis and the presentation of these data limit confidence in their ability to support the conclusions. Several control experiments appear to be lacking that could improve confidence. Specific concerns are noted below.

Major Points:
1. The most salient major conclusion of this manuscript is that certain receptors appear to follow a "use it OR lose it" model (gene expression and/or cell abundance increases with exposure to cognate odors) and others appear to follow a "overuse it AND lose it" model (gene expression and/or abundance decreases with exposure to cognate odors). The latter model is the focus of concluding Figure 6. Whereas this model may be true, the data in the manuscript support it only weakly, if at all. Specific reasons for this lack of confidence are:

1A: Double RNA-FISH experiments comparing animal exposure to male or female odors (Fig. 4c, 5f) are a critical component of this claim, but are evaluated using a semi-quantitative scoring system without any apparent statistical analysis. The images purportedly showing overlap between Egr1 and individual receptors are difficult to interpret given the thresholding scheme (i.e. hard to understand how they distinguished splotchy Egr1 signal from background). Without these data, the claim that

these experiments show that receptor changes are associated with constant exposure to cognate ligands is not supported.

1B: RNA-FISH images presented in Fig. 4B show that female mice exposed to male odors show dramatically increased Egr1 levels throughout the MOE compared to males exposed to female odors. This increased Egr1 staining probably increases the likelihood of encountering overlap between Egr1 and ANY OR (especially given the concerns about semi-quantitative scoring). It would be helpful if the authors performed the same analysis (altered to reflect concerns stated in 1A) using ORs that were NOT differentially expressed between SR and SF animals. If it turns out that 4/11 or 2/2 "hits" is significantly higher (e.g. by binomial test) than expected for any randomly-chosen receptor, this would support the authors' claims.

2. In the methods (lines 444-446), the authors describe the use of different thresholds for differential gene expression for ORs/VRs and all other genes. The justification for this choice is the apparently low abundance (assessed by FPKM values, which may not be the best metric) of ORs and VRs in these data sets. The authors then proceed to highlight that differentially expressed genes were enriched for ORs/VRs in MOE/VNO respectively. If the threshold were indeed different, the gene ontology analysis is not appropriate.

3. The Discussion is overly broad, and appears to claim that the data in the manuscript explains gene expression changes (both up- and down-regulation) in terms of sex or odor exposure. Comparisons are made between MOE and VNO (e.g., sex separation affecting MOE more than VNO) that are not cohesive. Whereas the overall conclusion may be true that odor exposure can drive changes in olfactory epithelial sensitivity (which is supported by previous literature), this discussion reads as a grab-bag of claims, some of which are not necessarily informed by the experiments in this manuscript.

4. The language "OVERuse it and lose it" (especially because the flipside is the less dramatic "use it or lose it") is not justified. There have been no experiments comparing odorant exposure strength or duration to the observed effects on gene expression.

Minor Points:
In the Introduction (lines 70-72) the authors leave out feces, which are also known sources of AOS ligands that differ based on sex (Doyle et al., 2016).

In the Results, lines 147-150, the assertion is made that the gene expression differences in the MOE and VNO are a result of specific changes to males and females, respectively. The logic supporting this claim is unclear from the data as presented.
The removal of pups in CF/CM conditions is understood to be a way to better control the long-term olfactory exposure of the test animals. However, this practice is almost certain to cause pulses of extreme stress for the females that may change their odorant profiles (i.e through the excretion of stress-associated urinary compounds, volatile alarm odors, etc.). This is worthy of discussion.

Experimental "Ns" for some experiments are missing (e.g. Fig. 4a). For RNA-FISH, the experimental N used for calculating standard error is not clear. If n = 4 mice and 5 sections are counted per mouse, is the total for the statistical test 4 or 20? If the latter, this may overestimate effect sizes.
Heat map presentation of differential expression (e.g. Fig. 2d, 5a, etc) lack a color scale ("colormap") explaining their meaning.

The total number of mice used for these experiments should be stated in the Methods.

Reviewer #3:
Remarks to the Author:
This is an interesting manuscript by Santoro and colleagues showing that housing male and female mice together or separately changes the expression frequency of certain chemoreceptors, Olfactory (OR) and Vomeronasal (VR) receptors. The transcriptional differences are convincing and are supported by RNA FISH experiments suggesting that in some cases represent differences in frequency of expression. The finding is important and interesting and constitutes an additional piece of evidence in the growing literatures suggesting that the odorant environment affects the expression profiles of chemoreceptors. There are a couple of concerns to be addressed.
1) Separating male from female mice indeed changes their odorant environments however, there are other physiological changes linked to the separate/combined housing of male and female mice. For example, when male and female mice are co-housed, they mate, have pups etch. It is possible that lifestyle differences produced by housing conditions influence the expression of the OR and VR repertoire (for example, hormone levels between the two housing conditions may be significantly different). To make a convincing argument for the odorant environment, the authors should perform experiments where male and female mice are housed separately but exchange odorants (i.e. have an odor permeable separator preventing males interacting with females while they can still smell them).
2) Because the expression frequency of OR genes changes significantly from section to section, the counting of the number of OR expressing cells should be presented as number of positive cells/OE section.
3) The authors have previously showed that a histone variant expressed in an activity dependent fashion influences the lifespan of these neurons. It would be interesting to explore whether this process is involved in the current observations. However, this is not a crucial experiment and if this would delay publication for 6 moths or more it is not a necessary experiment for this study.

Reviewers' comments:

Reviewer #1 (Remarks to the Author):

Linden et al tested the effects of the social context (being in the presence of same sex only conspecifics or mixed gender) on the cellular and molecular properties of the mouse olfactory system (from weaning until 6 months of age). Therefore, they had sex-separated females (SF), sex-separated males (SM) and sex-combined males and females (CF and CM). They analyzed the MOE, VNO and OB of the manipulated animals by RNAseq and histology (including using IEGs to test the responsiveness of OSN/VSN whose OR/VRs were differentially expressed).

The authors found more differentially expressed genes between SF and SM than between CF and CM. Chemoreceptors made up a large percentage of the differentially expressed genes and the differential expression was correlated with the number of sensory neurons expressing the receptor. Finally, they also show that many of the differentially expressed receptors detect male or female specific odors. These results were previously shown in the MOS, but the authors extend it to the vomeronasal system.

Response 1-1:
We agree that our study extends previous findings of experience-dependent sensory neuron population changes to the VNO, but believe that the novelty of our study goes beyond that for the following reasons: 1) To our knowledge, this is the first investigation of the scope of changes within the olfactory system as a function of exposure to or isolation from odors of the opposite sex, which are highly salient. 2) This is the first study to demonstrate that sex-separation can induce significant sex-specific differences in the makeup of the OSN population. 3) This study has identified ORs and VRs that are candidates for receptors that detect sex-biased odors.

The authors claim that the differences they find in the olfactory system are due to olfactory scene created by the sex separation and that these differences probably arise by the loss of specific SN (when they are stimulated by specific odors in the combined group).

The study is a small tour de force and seems well done to me (as far as I understand, there were 6 samples for each condition and in the SF/SM group these come from 3 independent cages and in the CF/CM they come from 6 independent cages; it would be nice to have the full experimental design in the supplementary information, including the origin of the animals: are there siblings between animals distributed through the different cages?). Founder effects could interfere with the expression of chemoreceptors.

Response 1-2:
We agree with the reviewer that additional details about sample preparation would be helpful. We have therefore added an additional figure (Supplementary Figure 1) to summarize the number of mice and tissue samples used to generate RNA samples, as well as how RNA samples were pooled to generate the 36 RNA-seq libraries used in this study. We have also added additional details to the Methods section of the revised manuscript.

As now described in the Methods section (page 19), we set up sex-separated and sex-combined cages by weaning mice into cages together with their siblings or, when necessary, with age matched mice from different litters. This was done so that all mice in a given cage would be age-matched to enable their addition to experimental cages and removal for euthanasia on the same dates as their cage-mates, thus maximizing consistency with respect to the developmental status and experiences of mice within each sex/condition group (SF, SM, CF, CM). To generate the RNA pools used to prepare the 36 RNA-seq libraries used in the study, we prepared RNA from 108 individual tissue samples (MOE, VNO, OB) and pooled equal quantities of 3 RNA samples extracted from tissues obtained from 3 individual non-sibling mice that had been housed in 3 different cages. The pooling of samples from different litters/cages was done to minimize the possibility of any cage- or litter-specific effects on experience and gene expression. We believe that this addresses the reviewer's concern regarding founder effects.

However, similar studies have been done previously, in particular I would like to emphasize the study by Ibarra-Soria et al, eLife 2017. Very similar results were observed for the MOE. In this paper the manipulations were different (embryos were transferred at very early stage between mothers, animals were cross-fostered or exposed to artificial odorants), but the observed phenomena was the same: changes in the expression of chemoreceptors and the correlated abundance of OSN expressing the receptor. In the current study, the authors claim to perform a manipulation where the animals were exposed to a different socially driven olfactory experience. It is true that they find differences in the composition/organization of the olfactory system. However, I do not think they can argue about what drives the observed changes.

Response 1-3:

a. We agree that findings of our study corroborate those of previous studies, but feel that it also uncovers new and interesting results that have not yet been reported. As noted in response 1-1(above), our study analyzes the results of exposure to or isolation from odors of the opposite sex, which are highly complex and salient. In contrast, the odors in the Ibarra-Soria paper (which is referenced multiple times in our manuscript) used single or small numbers of artificial compounds. Also, as noted in Response 1-1, we believe that our study goes beyond showing that olfactory experience causes changes in OSN abundance to show that the unique olfactory experiences of sex separation contribute to the generation of substantial sex-specific differences in the MOE and, to a lesser extent, the VNO. Our study also identifies candidate OR and VRs that detect sex-specific or sex-biased odors.
b. The last point (what drives observed changes) is addressed in Response 1-5 (below).

The 4 experimental groups of this manuscript cannot be compared. SF housed for 6 months will get along well. In contrast, SM housed for 6 months will fight and establish hierarchy (I am actually surprised they could maintain 4 males in a cage for 6 months in a healthy condition; even if the males are sexually naïve, in my lab we start observing aggressive encounters post puberty and males must be separated to prevent barbering, biting and other aggressions).

Response 1-4:
We thank the reviewer for this important question. It is true that male mice housed together as adults can fight. To guard against this possibility, we and members of our facility's professional animal care staff monitored the mice (both visually and via auditory cues) for any signs of aggression, typically checking 3-4 times per day. We observed no evidence of fighting among any of the cohorts, including the sex-separated males. We believe that this is because we used mice of the C57Bl/6 strain, part of the Mus *musculus musclus* subspecies, which were housed together without interruption from weaning (and in many cases, from birth) until euthanasia. Male mice of this strain are known to be capable of living together indefinitely from weaning without fighting or the formation of hierarchies (Bisazza, 1981; Kappel et al., 2017). Thus, the ability to stably house males together is strain-dependent. Information about male-male aggression has been added to the Results (page 5) and Methods (page 19) sections of the revised manuscript.

Finally, in the CF/CM group, animals had sex, females went through pregnancy an delivery several times. The neuroendocrine state of these animals is completely different and therefore I do not think we can pinpoint any cause to the observed results. For example, Shingo et al, (Shingo et al 2003. Pregnancy-stimulated neurogenesis in the adult female forebrain mediated by prolactin. Science) showed that neurogenesis changes through pregnancy and even mating. The odor context is different indeed, but the behaviour of the animals is even less comparable. Also, for example, the stress level of some of the SM in the sex-separated group must have been quite high. Even though the influence of stress hormones on the OE is controversial, it seems like chronic exposure to cortisol has an effect of the OB according to Siopi et al, J Neuro 2016. The same can be extended to sex hormones, which are not comparable across the different groups.

Response 1-5:
We appreciate the reviewer's perspective and agree that there is a need to be nuanced in how we interpret the results of our study. As described and now expanded upon in the Results (page 12) and Discussion (pages 16-17) sections of the revised manuscript, we acknowledge the possibility that sex-specific endocrine effects could contribute to sex-specific differences observed between mice in the four different experimental groups. Nevertheless, we feel that there is compelling evidence that the odor environments between experimental groups play at least some role in driving observed differences. This conclusion is based on the following lines of reasoning:

a. As the reviewer rightly points out, a mouse's endocrine status may be sex-specifically affected when housed in a sex-combined environment, perhaps as a result of mating and pregnancy. Moreover, such sex-specific hormone differences might be expected to cause a greater number of sex-specific gene expression differences in sex-combined mice than in sex-separated mice, as the former presumably experience greater sex-specific hormone effects. However, <u>we actually see significantly fewer</u> sex-specific differences in the sex-combined mice compared to the sex-separated mice. These results suggest that endocrine effects of housing males and females together are likely not the major driver of observed olfactory differences.
b. Our IEG results provide strong circumstantial evidence that at least some of the observed OR expression differences may be driven by odor experience.
c. Since the initial submission of our manuscript, we have performed experiments showing that disrupting olfactory activity via unilateral naris occlusion (UNO) alters the abundance of sex-

biased ORs. For example, UNO significantly increases the abundance of Olfr912-expressing OSNs on the closed side and decreases the abundance of Olfr1419-expressing OSNs on the closed side. These new findings corroborate our OR expression and IEG results, which indicate that Olfr912 abundance is decreased by exposure to male odors and Olfr1419 abundance is increased by exposure to male odors. Thus our new UNO results support the hypothesis that differences in odor experience play a role in driving expression differences in ORs that differ in abundance between sex-separated male and female mice. A new figure (Fig. 6) has been added to the revised manuscript to exhibit these data. Corresponding text has also been added to the Results section (pages 12-13).

Despite the lines of evidence listed above, we cannot completely rule out the possibility that endocrine effects may play a role in some of the sex-specific differences observed. This point has been emphasized in the Discussion section of the revised manuscript (pages 16-17). Moreover, we have softened the language used throughout the manuscript (e.g., see the last sentence of the Abstract) in order to convey that sex-specific differences are likely driven, at least in part, by the distinct odor experiences of sex-separated male and female mice.

So, my point is the following: if you want to just claim that animals that experience different social environment have different olfactory system, I think that is reasonable. But that has been shown before several times. In contrast, if the goal was to pinpoint the differences to the olfactory environment, I do not think it is possible with the experimental design used in this study. There are so many different variables that are not comparable across the groups that it is impossible in my view to do it. I acknowledge that perfect experiment (having sex-separated and sex-combined mice cohabiting and performing the same behaviour is literally impossible). But in this case, maybe a simpler exposure to bedding (or even full animals separated by a perforated wall where mice could exchange volatile and non-volatile cues) would make it possible to attribute the changes to the olfactory scene.

My main concerns are therefore with the main claim of the ms. I do not think the authors can claim that "Sex separation induced differences that appear to result from the fact that male and female mice emit distinct chemical profiles" even if some of the differences are observed in OR/VR that specifically respond to male/females cues.

Response 1-6:
As described in Response 1-5 (above), we think that our results provide compelling evidence that odor experience contributes to sex-specific differences in the MOE. Moreover, we think that our findings that sex separation induces sex differences in the olfactory system would be of interest regardless of mechanism, as they might provide insights into the causes of sex-specific differences in olfactory function, which have been reported in multiple studies. To our knowledge, the phenomena described in the present study have not been reported to date.

Despite the IEGs results, the authors do not test the biological relevance of the differential expression: for example, do SF have higher discriminability power than CF? The authors could test the sex discrimination capacities of SF versus CF.

Response 1-7:

a. Because we observed both male- and female-biased changes in the abundance of distinct OR-expressing OSNs in gender separated mice, it is difficult to predict how this would affect sex discrimination in sex-separated compared to sex-combined mice. Moreover, the attribution of any observed sex-odor discrimination differences to MOE/VNO changes as a result of sex separation may be difficult to tease out from changes elsewhere in the olfactory pathway (e.g., olfactory cortex) that might affect sex discrimination (e.g., due to the association of sex-specific odors with reward).
b. We think that addressing the biological role of the observed changes will be possible following the identification of the sex-specific ligands that are detected by the altered ORs. We could then use this information to identify how the presence or absence of the odors affects discrimination. In our view, such experiments are beyond the scope of this manuscript.

Reviewer #2 (Remarks to the Author):

The manuscript by Van der Linden et al. examines the long-term effects of environment (housed by sex or co-housed) on gene expression changes in mammalian olfactory systems. The authors use a combination of RNA-seq and RNA-FISH on olfactory tissues, finding evidence for sex- and experience-dependent changes in gene expression. The authors provide detailed analysis of specific genes, focusing on chemosensory receptors in the main olfactory and vomeronasal epithelia. Two-color RNA-FISH was used to investigate odor sensitivities of differentially expressed chemosensory receptors, finding that some differentially expressed genes appear to be enriched following environmental exposure to cognate odors, whereas other differentially expressed genes appear to be diminished in the presence of cognate odors. In general, the data are clearly presented, but several concerns about analysis and the presentation of these data limit confidence in their ability to support the conclusions. Several control experiments appear to be lacking that could improve confidence. Specific concerns are noted below.

Major Points:
1. The most salient major conclusion of this manuscript is that certain receptors appear to follow a "use it OR lose it" model (gene expression and/or cell abundance increases with exposure to cognate odors) and others appear to follow a "overuse it AND lose it" model (gene expression and/or abundance decreases with exposure to cognate odors). The latter model is the focus of concluding Figure 6. Whereas this model may be true, the data in the manuscript support it only weakly, if at all. Specific reasons for this lack of confidence are:

1A: Double RNA-FISH experiments comparing animal exposure to male or female odors (Fig. 4c, 5f) are a critical component of this claim, but are evaluated using a semi-quantitative scoring system without any apparent statistical analysis. The images purportedly showing overlap between Egr1 and individual receptors are difficult to interpret given the thresholding scheme (i.e. hard to understand how they distinguished splotchy Egr1 signal from background). Without these data, the claim that these experiments show that receptor changes are associated with constant exposure to cognate ligands is not supported.

Response 2-1:
We thank the reviewer for this excellent suggestion. We have now presented the Egr1 data quantitatively and have analyzed it statistically in revised versions of Figure 4, Figure 5, and Supplementary Figure 4. We believe that these changes clarify our findings.

1B: RNA-FISH images presented in Fig. 4B show that female mice exposed to male odors show dramatically increased Egr1 levels throughout the MOE compared to males exposed to female odors. This increased Egr1 staining probably increases the likelihood of encountering overlap between Egr1 and ANY OR (especially given the concerns about semi-quantitative scoring). It would be helpful if the authors performed the same analysis (altered to reflect concerns stated in 1A) using ORs that were NOT differentially expressed between SR and SF animals. If it turns out that 4/11 or 2/2 "hits" is significantly higher (e.g. by binomial test) than expected for any randomly-chosen receptor, this would support the authors' claims.

Response 2-2:
We appreciate this excellent suggestion. We have now included analyses of 3 negative control ORs and 3 negative control VRs that do not significantly differ in their expression (according to RNA-seq) between SF and SM samples. As now presented in the Results section of the revised manuscript (pages 9-10, 12), none of the negative control receptors tested showed significant differences in co-expression with Egr1 following exposure to male or female mice. Using a *z*-score test for two population proportions, we found that the proportion of sex-biased ORs that are significantly responsive to either male or female mice (7 of 13 tested) is significantly ($p = 0.046$) higher than that of the negative control ORs (0 of 3). This result suggests that the positive responses observed were not due to chance. A similar test for whether the proportion of sex-biased VRs that are responsive to either male or female mice (3 of 6 tested) is higher than that of the negative control VRs (0 of 3) gave a p-value of 0.067. While not statistically significant, the VR results are further supported by the observation that 4 of the 12 VRs that we identified as differentially expressed between SF and SM samples were previously shown in other studies to respond to sex-specific odors (Fig. 5f). Taken together, these results provide further confidence that the positive Egr1 results reflect OSN/VSN activity in response to exposure to male or female mice.

2. In the methods (lines 444-446), the authors describe the use of different thresholds for differential gene expression for ORs/VRs and all other genes. The justification for this choice is the apparently low abundance (assessed by FPKM values, which may not be the best metric) of ORs and VRs in these data sets. The authors then proceed to highlight that differentially expressed genes were enriched for ORs/VRs in MOE/VNO respectively. If the threshold were indeed different, the gene ontology analysis is not appropriate.

Response 2-3:
We are grateful to the reviewer for pointing out the inconsistency in how we described the statistical significance thresholds used in our study. As is now clarified in the RNA-seq (page 22) and newly added Statistics (page 25-26) subsections of the Methods section, we used an FDR of 0.05 ($q < 0.05$) as a significance threshold for identification of overall gene expression differences, including those used in gene ontology analyses (Fig. 2c). For analyses involving ORs and VRs alone, we used a

significance threshold of $p < 0.01$ (unadjusted), which enabled consideration of ORs/VRs that do not meet the $q < 0.05$ significance threshold due to low transcript abundance, but may nevertheless exhibit differential expression. Indeed, our RNA-FISH analyses confirmed that ORs/VRs that met the $p < 0.01$ threshold but not the $q < 0.05$ threshold are in fact differentially represented in SF and SM mice. We have marked the receptors in Figs. 2d, 5a and Suppl. Figs. 2c-e, 6c-d according to the significance threshold that they meet for differential expression by RNA-seq.

3. The Discussion is overly broad, and appears to claim that the data in the manuscript explains gene expression changes (both up- and down-regulation) in terms of sex or odor exposure. Comparisons are made between MOE and VNO (e.g., sex separation affecting MOE more than VNO) that are not cohesive. Whereas the overall conclusion may be true that odor exposure can drive changes in olfactory epithelial sensitivity (which is supported by previous literature), this discussion reads as a grab-bag of claims, some of which are not necessarily informed by the experiments in this manuscript.

Response 2-4:
We sincerely appreciate this feedback. In the revised version of the manuscript, we have consolidated and simplified the Discussion section. As part of this effort, we have removed the section about why sex separation affects the MOE to a greater extent than the VNO.

4. The language "OVERuse it and lose it" (especially because the flipside is the less dramatic "use it or lose it") is not justified. There have been no experiments comparing odorant exposure strength or duration to the observed effects on gene expression.

Response 2-5:
This is a good point. We have changed this throughout the revised version of the manuscript.

Minor Points:
In the Introduction (lines 70-72) the authors leave out feces, which are also known sources of AOS ligands that differ based on sex (Doyle et al., 2016).

Response 2-6:
This is an excellent point. We have added this reference to the revised version of the manuscript.

In the Results, lines 147-150, the assertion is made that the gene expression differences in the MOE and VNO are a result of specific changes to males and females, respectively. The logic supporting this claim is unclear from the data as presented.

Response 2-7:
We appreciate this feedback. Our intention was to indicate that both sexes undergo changes in gene expression due to sex-separation, but that males exhibit a slightly greater number of changes in the MOE and females exhibiting a greater number of changes in the VNO. We have clarified this in the Results section (page 7) of the revised manuscript.

The removal of pups in CF/CM conditions is understood to be a way to better control the long-term

olfactory exposure of the test animals. However, this practice is almost certain to cause pulses of extreme stress for the females that may change their odorant profiles (i.e through the excretion of stress-associated urinary compounds, volatile alarm odors, etc.). This is worthy of discussion.

Response 2-8:

1. We did not observe outward signs of stress (e.g., fighting, over-grooming) in adult mice following the removal of newborn pups from sex-combined cages, but acknowledge that there is a potential for elevated stress due to this manipulation. We therefore agree with the reviewer that this issue is worthy of discussion. We have addressed the implications of this possibility in the Discussion section (page 16) of the revised manuscript.
2. Related to our response 1-5, any stress responses due to the removal of pups from the sex-combined cages would most likely cause more sex-specific gene expression differences in sex-combined mice than sex-separated mice. However, in fact we observed fewer sex-specific differences in sex-combined mice.

Experimental "Ns" for some experiments are missing (e.g. Fig. 4a). For RNA-FISH, the experimental N used for calculating standard error is not clear. If n = 4 mice and 5 sections are counted per mouse, is the total for the statistical test 4 or 20? If the latter, this may overestimate effect sizes.

Response 2-9:
We are grateful to the reviewer for pointing out our omission of sample number for Fig. 4a (this figure panel has now been moved to Suppl. Fig. 5). We have added sample number information to the figure legend and have checked that sample numbers have been included throughout the manuscript. We agree that the basis of sample number for statistical analysis the RNA-FISH OSN/VSN frequency quantification experiments was unclear; we have clarified this in the revised manuscript. The sample numbers for standard error and P-value calculations for the OSN/VSN frequency quantification graphs and tables in Suppl. Figs. 2f and 6f refer to the number of different mice analyzed, with the value for each mouse determined by averaging results from 5 different tissue sections. In addition, in order to provide variance information for OSN/VSN frequencies across tissue sections, the tables in the revised versions of Suppl. Figs. 2f and 6f now also include (in parentheses) P-values based on treating each section as a separate sample.

Heat map presentation of differential expression (e.g. Fig. 2d, 5a, etc) lack a color scale ("colormap") explaining their meaning.

Response 2-10:
We are grateful to the reviewer for pointing out our omission of color scales and have added them to all relevant figures within the revised manuscript.

The total number of mice used for these experiments should be stated in the Methods.

Response 2-11:
We agree with the reviewer that this information is helpful and have included within the Methods section (page 19) of the revised manuscript a detailed accounting of the mice used in this study. We also have added a new figure (Suppl. Fig. 1) that describes the number of animals and tissue samples

used to generate RNA-seq libraries.

Reviewer #3 (Remarks to the Author):

This is an interesting manuscript by Santoro and colleagues showing that housing male and female mice together or separately changes the expression frequency of certain chemoreceptors, Olfactory (OR) and Vomeronasal (VR) receptors. The transcriptional differences are convincing and are supported by RNA FISH experiments suggesting that in some cases represent differences in frequency of expression. The finding is important and interesting and constitutes an additional piece of evidence in the growing literatures suggesting that the odorant environment affects the expression profiles of chemoreceptors. There are a couple of concerns to be addressed.
1) Separating male from female mice indeed changes their odorant environments however, there are other physiological changes linked to the separate/combined housing of male and female mice. For example, when male and female mice are co-housed, they mate, have pups etch. It is possible that lifestyle differences produced by housing conditions influence the expression of the OR and VR repertoire (for example, hormone levels between the two housing conditions may be significantly different). To make a convincing argument for the odorant environment, the authors should perform experiments where male and female mice are housed separately but exchange odorants (i.e. have an odor permeable separator preventing males interacting with females while they can still smell them).

Response 3-1:

a. We have addressed similar issues raised by reviewers 1 and 2 in Responses 1-5 and 2-8. To reiterate one of the key points stated above, sex-specific hormone changes due to experiences such as mating and pup birth would presumably cause a greater number of sex-specific differences under sex-combined conditions, where these experiences occur. However, our results show the opposite effect: we observed significantly <u>fewer</u> sex-specific differences in gene expression in sex-combined mice compared to sex-separated mice. Nevertheless, we acknowledge that our results cannot rule out the possibility that at least some of the differences observed may be due to sex-specific hormonal effects. As noted above, we have expanded the discussion of this possibility in the revised manuscript (pages 16-17) and have softened the language used throughout the manuscript (e.g., see the last sentence of the Abstract) in order to convey that sex-specific differences are likely driven, at least in part, by the distinct odor experiences of sex-separated male and female mice.
b. We think that repeating the experiment in our study using an odor-permeable separator is an interesting idea, as it could provide verification that hormonal changes due to mating and pub birth do not contribute substantially to observed sex-specific differences, particularly those observed in sex-combined mice. However, we have not repeated the experiment with the proposed modification for four reasons: 1) We believe that this modification would not be able to completely rule out sex-specific hormonal effects due to odor or other sensory information that would be exchanged as a result of housing mice in close proximity. 2) As noted above, the majority of sex-specific gene expression differences observed in this study were found in <u>sex-separated</u> mice, which would not be affected by the proposed modification. 3) Repeating the experiment with the proposed modification would require at

least an additional year to perform, including the time required to set up mating cages, generate mice, house mice until 6 months of age, perform dissections, and perform gene expression analyses. Thus, while this experiment may be informative, we feel that it is beyond the scope of this study. 4) In our view, the unilateral naris occlusion (UNO) experiments that we have added to the revised manuscript (Fig. 6; pages 12-13) address the reviewer's concern to some extent. These experiments demonstrate that olfactory deprivation causes differences in the expression frequencies of sex-biased ORs on the two sides of the MOE. These results support the conclusion that at least some of the sex-specific receptor expression differences observed following sex-separation are driven by differences in olfactory experience.

2) Because the expression frequency of OR genes changes significantly from section to section, the counting of the number of OR expressing cells should be presented as number of positive cells/OE section.

Response 3-2:
We agree with the reviewer that OR frequencies (specific OR-expressing cells/unit area) can vary from section to section. However, we have found that there can be at least as much variability in the number of OR-expressing OSNs across sections due to variations in neuroepithelial area from section to section. For example, anterior and posterior sections are typically smaller in total epithelial area compared to medial sections. (This is also true for VR expression within the VNO.) Although it requires more work (e.g., co-staining for Omp and measurement of the area of OMP-expression in each section), we have found that normalizing OR numbers within a section to neuroepithelial area (i.e., area of Omp expression) can be helpful for comparing the abundance of specific OR-expressing OSNs between different animals. Since a mouse's expression frequency for a specific OR should be proportional to its total numbers of OSNs that express that OR (the quantity that we are interested in), we think that this is a valid way to express the data. It is also an approach that has been used previously in numerous studies (e.g., Lee et al., 2009; Santoro and Dulac, 2012; Tian and Ma, 2008; Zhang et al., 2016). On the other hand, we are aware of other studies that have quantified OR expressing cells in terms of the number of specific OR-expressing OSNs/ section (uncorrected for neuroepithelial area). In our view, each of these approaches is valid and may have advantages.

3) The authors have previously showed that a histone variant expressed in an activity dependent fashion influences the lifespan of these neurons. It would be interesting to explore whether this process is involved in the current observations. However, this is not a crucial experiment and if this would delay publication for 6 moths or more it is not a necessary experiment for this study.

Response 3-3:
This is an interesting idea (one that we have considered and hope to address in the future)! Due to the time that would be required to complete this experiment (more than one year), however, we feel that it is beyond the scope of the present study.

Reviewers' Comments:

Reviewer #1:
Remarks to the Author:
The revised manuscript submitted by Linden et al contains substantial changes that address most issues raised by the three reviewers. The manuscript includes a new experiment, new analyses and a significant amount of re-writing.

I am satisfied by the revised version and appreciate the effort made by the authors. The occlusion experiment indeed corroborates their findings; the toning down of the findings, addition of other possible explanations for the results, expansion of the methods and focusing of the discussion, make the manuscript easier to read and better in my opinion.

I think the current manuscript is suitable for publication.

Reviewer #2:
Remarks to the Author:
The revised manuscript by van der Linden, et al. is much improved compared to the original submission, and the authors should be lauded for the improvements. The unilateral naris occlusion experiments are a useful addition to the manuscript and generally support the conclusions, even though the results are not entirely as expected. Clarifying the experimental procedures and statistical analysis made a marked improvement in the overall presentation.

One major issue remains:

The addition of data from ORs that were not differentially expressed by RNA-seq analysis is important for establishing the validity of the approach (Lines 268-271 and Response 2-2), but there are issues with (A) the negative control sample number and (B) the "z-score test" used to support the claim.

Starting with (B), can the authors please explain how one uses a "z-score test" to estimate a p-value on these binary outcomes? What was actually done is entirely unclear. The suggestion was to utilize a simple binomial test. The problem is that with 0/3 in the negative control set, one cannot use a 0% rate in the binomial test as the probability of observing a "false positive" by RNA-FISH by chance in the test groups. Substituting an arbitrary low probability based on the 0/3 observation would be perilous. However, one could use the positive results (7/13 ORs and 3/6 VRs) as an expected rate of discovery in a "positive control" group and then test the outcome of the negative control group (i.e. treating it as the test group). For the 7/13 ORs (54% expected rate), the equivalent binomial probability of observing 0 differences by RNA-FISH in 3 tests if the negative and positive control groups were equal is 0.0983 (i.e. "binocdf(0,3,7/13)"). For VSNs, this would be 0.125 (i.e. "binocdf(0,3,3/6)").

This brings up (A). By choosing just 3 ORs to test as negative controls, there still isn't enough data to claim that the responses observed were not due to chance. Ideally, the authors would add equal numbers of positive and negative control samples (for both ORs and VRs). At a minimum they should test enough control ORs to be able to support their claim. For what it's worth, at N=6, if there were still zero RNA-FISH positives in the negative control group, the binomial test p-values would be 0.0097 for OSNs (i.e. "binocdf(0,6,7/13)") and 0.0156 for VSNs (i.e. "binocdf(0,6,3/6)").

*This seems quite technical, but one of the most critical claims of the paper is that bulk RNA-seq

analysis can be used to identify likely changes in the number of chemosensory neurons in the nose. This result will surely be cited in future RNA-seq based studies. If this claim is allowed to pass through without proper validation, it could become a major problem for the field.*

Minor point:
Lines 486-491 appear to be redundant with 569-573.

We are extremely grateful to the reviewers for taking the time to evaluate our manuscript a second time.

Reviewers' comments:

Reviewer #1 (Remarks to the Author):

The revised manuscript submitted by Linden et al contains substantial changes that address most issues raised by the three reviewers. The manuscript includes a new experiment, new analyses and a significant amount of re-writing.

I am satisfied by the revised version and appreciate the effort made by the authors. The occlusion experiment indeed corroborates their findings; the toning down of the findings, addition of other possible explanations for the results, expansion of the methods and focusing of the discussion, make the manuscript easier to read and better in my opinion.

I think the current manuscript is suitable for publication.

We sincerely appreciate these comments.

Reviewer #2 (Remarks to the Author):

The revised manuscript by van der Linden, et al. is much improved compared to the original submission, and the authors should be lauded for the improvements. The unilateral naris occlusion experiments are a useful addition to the manuscript and generally support the conclusions, even though the results are not entirely as expected. Clarifying the experimental procedures and statistical analysis made a marked improvement in the overall presentation.

We also sincerely appreciate these comments.

One major issue remains:

The addition of data from ORs that were not differentially expressed by RNA-seq analysis is important for establishing the validity of the approach (Lines 268-271 and Response 2-2), but there are issues with (A) the negative control sample number and (B) the "z-score test" used to support the claim.

Starting with (B), can the authors please explain how one uses a "z-score test" to estimate a p-value on these binary outcomes? What was actually done is entirely unclear. The suggestion was to utilize a simple binomial test. The problem is that with 0/3 in the negative control set, one cannot use a 0% rate in the binomial test as the probability of observing a "false positive" by RNA-FISH by chance in the test groups. Substituting an arbitrary low probability based on the 0/3 observation would be perilous. However, one could use the positive results (7/13 ORs and

3/6 VRs) as an expected rate of discovery in a "positive control" group and then test the outcome of the negative control group (i.e. treating it as the test group). For the 7/13 ORs (54% expected rate), the equivalent binomial probability of observing 0 differences by RNA-FISH in 3 tests if the negative and positive control groups were equal is 0.0983 (i.e. "binocdf(0,3,7/13)"). For VSNs, this would be 0.125 (i.e. "binocdf(0,3,3/6)").

We agree with the reviewer that the description of the statistical method that we used in the previous version of the manuscript was inadequate. To answer the reviewer's question, we applied what we should have referred to as a two-proportion z-test, which is described here: https://onlinecourses.science.psu.edu/stat414/node/268/. We envisioned that this test would enable statistical testing of whether the difference in the proportions of ORs/VRs that respond differentially to male- and female-specific odors in the *Egr1* assay is significantly higher among receptors differentially expressed between separated males and females compared to negative control receptors that are not differentially expressed. We agree with the reviewer, however, that the binomial test is perhaps better suited to addressing this question and thank the reviewer for describing in detail how it may be applied in this situation. In this revision, we have used the binomial test, as suggested.

This brings up (A). By choosing just 3 ORs to test as negative controls, there still isn't enough data to claim that the responses observed were not due to chance. Ideally, the authors would add equal numbers of positive and negative control samples (for both ORs and VRs). At a minimum they should test enough control ORs to be able to support their claim. For what it's worth, at N=6, if there were still zero RNA-FISH positives in the negative control group, the binomial test p-values would be 0.0097 for OSNs (i.e. "binocdf(0,6,7/13)") and 0.0156 for VSNs (i.e. "binocdf(0,6,3/6)").

We agree that having additional negative control receptors would strengthen our conclusion that the ORs/VRs that we have found to be differentially responsive to male- and female-specific odors in the *Egr1* assay were unlikely to have been identified by chance. We therefore prepared new *in situ* hybridization probes for 5 additional ORs and 3 additional VRs that are not differentially expressed between separated male and female mice by RNA-seq. Using these probes as negative controls in the *Egr1* assay, we found that none of these additional receptors responds differentially to male- and female-specific odors. To ensure that the *Egr1* assay was working as expected, we also included probes for an OR (Olfr912) and a VR (Vmn1r185) that we had previously shown to be responsive to sex-specific odors as positive controls.

This seems quite technical, but one of the most critical claims of the paper is that bulk RNA-seq analysis can be used to identify likely changes in the number of chemosensory neurons in the nose. This result will surely be cited in future RNA-seq based studies. If this claim is allowed to pass through without proper validation, it could become a major problem for the field.

We agree and appreciate the reviewer's pointing this out.

Minor point:
Lines 486-491 appear to be redundant with 569-573.

We thank the reviewer for pointing this out. We have removed lines 486-491.

Reviewers' Comments:

Reviewer #2:
Remarks to the Author:
All of my concerns have now been adequately addressed. The authors have greatly improved the manuscript from its initial submission.

Editorial requests:

* Nature Communications uses a transparent peer review system, where for manuscripts submitted from January 2016 we are publishing the reviewer comments to the authors and author rebuttal letters of our research articles online as a supplementary peer review file. Please let us know in the cover letter when submitting the final version of your manuscript if you wish to opt out of this scheme or not. If you are concerned about the release of confidential data, we do permit redactions in the interest of confidentiality. If you would like to remove such information from these files, then please let us know specifically what information you would like to have removed. Please note that we cannot incorporate redactions for other reasons. Reviewer names will be published in the peer review files if the reviewer comments to the authors are signed by the reviewer, or if reviewers explicitly agree to release their name. For more information, please refer to our FAQ page at:
https://www.nature.com/documents/ncomms-transparent-peer-review.pdf

As indicated in our cover letter, we do not wish to opt out of the transparent peer review system, nor are any redactions requested.

* Please ensure that an updated editorial policy checklist that verifies compliance with all required editorial policies is completed and uploaded with the revised article. All points on the policy checklist must be addressed; if needed, please revise your manuscript in response to these points. Please note that this form is a dynamic 'smart pdf' and must therefore be downloaded and completed in Adobe Reader.
Editorial policy checklist: https://www.nature.com/authors/policies/Policy.pdf
We have completed the editorial policy checklist.

* Your manuscript should comply with our policies and format requirements, detailed in our checklist for authors at:
https://www.nature.com/documents/ncomms-manuscript-checklist.pdf

We have made sure that we are in compliance with the policies and format requirements detailed in the checklist. As such, we have:

- Added full postal addresses for all authors
- Shortened the abstract to < 150 words
- Altered the abstract so that the results of the present study are written in the present tense
- Expressed unit dimensions in figure legends using negative integers
- Altered the last paragraph of the Introduction so that the results of the present study are written in the present tense
- Shortened subheadings in Results to less than 60 characters
- Changed 'Suppl.' to 'Supplementary'
- Removed subheadings from the Discussion section
- Shortened subheadings in Methods to less than 60 characters

- Reduced the number of references to 70
- Provided supplementary information as a separate PDF file

* Data availability statements and data citations policy: All Nature Communications manuscripts must include a section titled "Data Availability" as a separate section after the Methods section but before the References. For more information on this policy, and a list of examples, please see http://www.nature.com/authors/policies/data/data-availability-statements-data-citations.pdf
In particular, the Data availability statement should include:
- Accession codes for deposited data
- Other unique identifiers (such as DOIs and hyperlinks for any other datasets)
- At a minimum, a statement confirming that all relevant data are available from the authors
- If applicable, a statement regarding data available with restrictions
- If a dataset has a Digital Object Identifier (DOI) as its unique identifier, we strongly encourage including this in the Reference list and citing the dataset in the Data Availability Statement.

Our manuscript includes a Data Availability section.

* DATA SOURCES: We strongly encourage authors to deposit all new data associated with the paper in a persistent repository where they can be freely and enduringly accessed. We recommend submitting the data to discipline-specific, community-recognized repositories, where possible and a list of recommended repositories is provided here: http://www.nature.com/sdata/policies/repositories

If a community resource is unavailable, data can be submitted to generalist repositories such as figshare (https://figshare.com/) or Dryad Digital Repository (http://datadryad.org/). Please provide a unique identifier for the data (for example a DOI or a permanent URL) in the data availability statement, if possible. If the repository does not provide identifiers, we encourage authors to supply the search terms that will return the data. For data that have been obtained from publically available sources, please provide a URL and the specific data product name in the data availability statement. Data with a DOI should be further cited in the methods reference section.

Please refer to our data policies here: http://www.nature.com/authors/policies/availability.html

RNA-seq data associated with this study have been deposited in NCBI Sequence Read Archive and NCBI Gene Expression Omnibus.

* To ensure correct hyperlinking of the accession codes in your manuscript, please add the hyperlink or DOI in square brackets directly after the code throughout (for example, '5XRN [http://dx.doi.org/10.2210/pdb5XRN/pdb]', '1483958 [https://dx.doi.org/10.5517/ccdc.csd.cc1lt5m6]', 'SRP109982

[https://www.ncbi.nlm.nih.gov/sra/?term=SRP109982]’ or 'NQLW00000000 [https://www.ncbi.nlm.nih.gov/assembly/GCA_002312845.1/]').

We have added hyperlinks to the accession codes in our manuscript.

* Please check whether your manuscript or Supplementary Information contain third-party images, such as figures from the literature, stock photos, clip art or commercial satellite and map data. We strongly discourage the use or adaptation of previously published images, but if this is unavoidable, please request the necessary rights documentation to re-use such material from the relevant copyright holders and return this to us when you submit your revised manuscript.

Our manuscript does not contain third-party images.

* Please supply the main manuscript file in Microsoft Word or LaTeX format.

We have supplied the main manuscript file in Microsoft Word.

* Please remove the subheadings from the Discussion.

We have removed subheadings from the Discussion.

* Please avoid using speech marks around words or phrases to convey emphasis.

We have removed speech marks from the manuscript.

* Please ensure that italics are used for scalar variables, physical constants, species and gene names, and bold font is used for numbering chemical compounds. Italics and bold font should not be used for emphasis.

We have removed the italics that had been used for emphasis.

* In each Figure and Supplementary Figure where error bars are used, they must be defined, and the number of experimental replicates stated. One statement at the end of each figure is sufficient if the error bars are equivalent throughout the figure.

We have defined error bars and stated the number of experimental replicates in all figures.

* Wherever p-values are stated in the main text and figure legends, please also state the name of the statistical test.

We have added the names of statistical tests to the main text and figure legends.

* Where p-values are presented as symbols/letters, please ensure that all symbols/letters are defined in the relevant figure legend, together with the statistical test used.

We have ensured that symbols representing p-values have been defined.

* Please ensure that all colour scales are defined in either the figure or its associated legend.

We have defined the color scales used.

* Please ensure that the corresponding dot plots are overlaid in the bar charts.

We have overlaid dot plots onto bar charts.

* Please ensure the figures are cited in the order they appear in the text. If a figure needs to be cited out of order, please use the text 'see below'.

All figures are cited in the order that they appear in the text.

* Please define any new abbreviations, symbols or colours present in your figures in the associated legends, noting that these should be written out in words (blue circles, red dashed line, etc.) as symbols will not appear properly in the HTML text.

We have done this.

* We anticipate that for the length of your manuscript, around 70 references would be appropriate.

We have reduced our reference list to 70.

* Please make a 'Competing Interests' statement after the 'Author Contributions' section that refers to all authors. If there are no competing interests, please add the statement "The authors declare no competing interests."

We have done this.

* Please note that we do not reformat Supplementary Information files; they will be uploaded with the published article as they are submitted with the final version of your manuscript. Please check everything very carefully and remove any track changes from the file. Failure to adhere to our style guidelines will result in delays in production. The only sections we permit in the Supplementary Information file are Supplementary Figures, Supplementary Tables, Supplementary Methods, Supplementary Notes, Supplementary Discussion, Supplementary References.
We have done this.

* Each Supplementary Figure should be accompanied by a legend, which should be presented below the figure and may be up to 350 words, that refers to all panels within the figure, and a title that summarises the figure and does not refer to specific panels. This also applies to spectra, which should be labelled as Supplementary Figures.

We have done this.

* Your paper will be accompanied by a two-sentence editor's summary, of between 250-300 characters, when it is published on our homepage. Could you please approve the draft summary below or provide us with a suitably edited version.

The expression of the olfactory receptor repertoire adapts to the olfactory context. Here, the authors demonstrate that housing male and female mice together or separately induces changes in expression frequency of olfactory and vomeronasal receptors, some of which detect sex-specific odors.

Please use the following:
Olfactory experience can alter the relative abundance of neurons expressing specific chemoreceptors. Here, the authors demonstrate that the distinct odor experiences of sex-separated male and female mice induce sex-specific differences in the abundance of neurons that detect sexually dimorphic odors.

* As part of our efforts to communicate our content to a wider audience, we endeavour to highlight papers published in {$journal_name} on the journal's Twitter account (@NatureComms). If you would like us to mention authors, institutions or lab groups in these tweets, please provide the relevant twitter handles in your cover letter upon resubmission.

As indicated in our cover letter, we would like you to use the twitter handles @SSantoroLab and @DulacLab.

REVIEWERS' COMMENTS:

Reviewer #2 (Remarks to the Author):

All of my concerns have now been adequately addressed. The authors have greatly improved the manuscript from its initial submission.
